# CottonNet-MHA: a multi-head attention-based deep learning framework for cotton disease detection

**DOI:** 10.3389/fpls.2025.1664242

**Published:** 2025-11-21

**Authors:** Mostaque Md. Morshedur Hassan, Asmita Ray, Munsifa Firdaus Khan Barbhuyan, Mudassir Khan, Bayan Alabdullah, Md. Faruqul Islam, Barga Mohammed Mujahid

**Affiliations:** 1 Department of Computer Science and Engineering, Eudoxia Research University, New Castle (City of Newark), Delaware, DE, United States; 2 Swami Vivekananda Institute of Science & Technology, Department of Computer Science and Engineering, Kolkata, West Bengal, India; 3 Department of Information Technology, Assam Skill University, Mangaldai, Assam, India; 4 Department of Computer Science, College of Computer Science, Applied College Tanumah, King Khalid University, Abha, Saudi Arabia; 5 Jadara University Research Center, Jadara University, Irbid, Jordan; 6 Department of Information Systems, College of Computer and Information Sciences, Princess Nourah bint Abdulrahman University, Riyadh, Saudi Arabia; 7 Department of Computer Science and Engineering, Global Institute of Management & Technology, Nadia, West Bengal, India; 8 Department of Computer Science, Applied College Khamish Mushaiyt, King Khalid University, Abha, Saudi Arabia

**Keywords:** deep learning, CNN, transfer learning techniques, cotton plant, cotton disease, agriculture, Gradient-weighted Class Activation Mapping (Grad-CAM)

## Abstract

India is an agro-based country. The major goal of agriculture is to produce disease-free healthy crops. For Indian agronomists, cotton is a profitable commercial and fibre crop, it is the world’s second-biggest export crop after China. Cotton production is also affected in a negative way by high use of water, authority of soil erosion and the practice of using dangerous fertilizers and pesticides. The two greatest threats to the rapid growth of the crop are the sucking bugs and cotton diseases. Prompt detection and accurate identification of diseases is vital to ensure healthy crop growth and achieve better yields. The primary objective of this research is to build a model by implementing deep learning-based approaches to spot infections in cotton crops. Deep learning is used because of its exceptional results in classification and image processing tasks. To address this issue, we developed CottonNet-MHA a novel deep learning framework to identify pathological symptoms in cotton leaves. The model employs multi-head attention mechanisms to strengthen feature learning and highlight the diseased-affected regions. To evaluate the performance of the proposed model, five pretrained transfer learning architectures—VGG16, VGG19, InceptionV3, Xception, and MobileNet were used as benchmark models. Furthermore, Gradient-weighted Class Activation Mapping (Grad-CAM) visualization was applied to enhance the trustworthiness and interpretability of the model. A web-based application was developed to deploy the trained model for real-world applicability. The performance analysis is carried out on the developed model based on the conventional models and the results indicate that CottonNet-MHA dominates the conventional models with respect to its accuracy as well as efficiency in the detection of diseases. The use of attention mechanisms approach strengthens the model’s diagnostic accuracy and overall reliability. Grad-CAM results further demonstrated that the model effectively targets diseased areas, enhancing interpretability and reliability. Discussion: The study shows that CottonNet-MHA not only automates disease detection but also enhances interpretability through Grad-CAM analysis. The developed web platform allows the model to be applied in real-world environments, supporting live disease monitoring. The proposed framework not only improves the accuracy of cotton disease diagnosis but also offers potential for extension to other crop disease detection systems.

## 1 Introduction

Around 70% of individuals residing in rural and semi-urban regions rely heavily on agricultural resources and agriculture is the key origin of their livelihood (Arjun, 2013). Cotton is a cash crop that has a big impact on India's economy. Due to India's varied environment, farmers cultivate a range of crops, including horticulture, cash crops, food crops, plantations, and numerous others (Rajasekar et al., 2021). In India, the agriculture sector has a big influence on the economy. Prompt detection and assessment of crop infections is very crucial in agriculture operations (Vallabhajosyula et al., 2022).

Every year, farmers suffer significant financial losses due to crop disease. Consequently, prompt, accurate, and timely disease identification reduces product loss and enhances product quality. Consequently, it contributes to the nation's economic expansion (Shrivastava et al., 2019). Various computer vision algorithms are available to identify plant diseases effectively (Wang et al., 2017). In contemporary agriculture, there are two distinct phases: the first phase, which covers the years 1943 to 2006, and the second phase, which began in 2012 and primarily applies deep learning ideas for detection. During the early phase of neural network evolution, key techniques such as Backpropagation, Chain Rule-based learning, and the Neocognitron architecture were developed (Saleem et al., 2019). Deep Learning (DL) methods like AlexNet, ResNet, Segnet, YOLO, UNet, and Fast R-CNN were implemented in the second stage (Shrivastava and Pradhan, 2021; Pandian et al., 2022). Deep learning algorithms have become the driving force behind modern AI-based computer vision, offering a significant advancement over traditional machine learning methods that relied heavily on manually crafted feature extraction. These advanced models learn to extract relevant features directly from raw data.

Recent advancements in remote sensing, computer vision, and AI have significantly enhanced the capability to analyze complex image data across diverse domains. Studies have demonstrated innovative applications of deep learning in remote sensing, terrain mapping, and environmental monitoring (Xu et al., 2023; Wang et al., 2024; Lu et al., 2025; Tu et al., 2024; Gong et al, 2024). These developments collectively emphasize the growing potential of AI-driven models for image-based agricultural analysis, such as cotton disease detection.

Many researchers employed well-known deep learning architectures for plant disease classification, including AlexNet, VGGNet, InceptionV3, ResNet, and DenseNet (Nigam et al., 2023; Too et al., 2018). Deep neural approaches are extensively utilized in diverse applications like image processing, autonomous driving, healthcare, and more.

One notable limitation of deep neural architecture is its reliance on large volumes of data for effective model training. When datasets contain only a small number of images, the model's performance tends to suffer. Transfer learning offers a solution to this issue, as it allows networks to be trained with significantly less data. Transfer learning facilitates the learning of a new task by utilizing the information acquired from a related prior task. Its key advantages include optimized training process, better generalisation performance, lowers resource consumption and simplifies the development process of deep learning systems (Hassan et al., 2021; Pandian et al., 2022).

In recent decades, extensive research efforts have focused on identifying various Phytopathological conditions, resulting in the development of several deep learning-based models to analyse images (Wang et al., 2019). Due to the usage of laboratory conditioned dataset, this work is currently experiencing a significant problem. The images in this case did not generalize to real field images because they were generated in a lab for training and assessment purposes. Model performance was significantly impacted by the high complexity of real plant images. The selection and extraction of features are challenging, as overlapping features in images complicate their identification (Krishnakumar and Narayanan, 2019). Identification of the plant disease at an early stage is important to prevent from spreading rapidly. Farmers apply poisonous drugs to control diseases in plants. We consume these harmful drugs into our daily routine. The use of dangerous chemicals in agricultural operations can be greatly decreased or perhaps eliminated with early diagnosis of plant diseases.

The remaining sections of the paper are organized as follows: Literature Review, Proposed methodology, Classification models, Data set and implementation details, Results and Discussion, Responsive Web Application Development,GRAD-CAM Analysis,Comparative Analysis, Computational Efficiency and Resource Utilization, and Conclusion and Future Scope.

## 2 Literature survey

The accuracy of deep learning models has significantly increased because of the widespread application of transfer learning in computer vision, especially for identifying diseased cotton plants. A preliminary literature review was conducted to examine existing studies and methodologies in plant disease detection, focusing on models, objectives, research gaps, findings, limitations, and future scope. A summary of the reviewed literature is presented in the **Table 1**. The objective of this review was to evaluate various approaches, techniques, and algorithms employed by researchers in this domain. Notably, a large amount of research has demonstrated how transfer learning can improve the detection of diseased cotton crops when deep learning frameworks are used.

**Table 1:** Summary of the literature review.

Study	Model	Objective	Results
Rastogi et al., 2024	CNN	Classify diseases in potato, tomato & pepper for timely treatment	CNN achieved 86.21% accuracy on Plant Village dataset; supports yield & sustainability.
Zhou et al., 2024	REM- ShuffleNetV2	Lightweight model for crop leaf disease detection in real- field conditions.	Achieved 96.72% accuracy & 96.62% F1; better than DenseNet121 & EfficientNet; efficient & robust.
Islam et al., 2023	VGG-16, VGG- 19, Inception-V3, Xception	Transfer Learning models for cotton leaf disease detection & web-based real-time prediction	Xception achieved 98.7% accuracy; effective for disease diagnosis & scalable web deployment.
Zhang and Wang, 2023	BERT-BiGRU- CapsNet with Attention Pooling (BBGCAP)	Classify agricultural queries on crop diseases & pests	Achieved >90% precision, recall, F1; efficient for real-time Q&A.
Borhani et al., 2022	Vision Transformer (ViT)	Lightweight DL model for real-time plant disease classification	Outperforms CNNs in F1, recall, precision with fewer parameters; balances accuracy & speed.
Zhu et al., 2022	VGG16, ResNet164, DenseNet40	Optimize cotton disease detection using pruning, transfer learning & model compression for mobile devices	Pruned DenseNet40 achieved 97.23% accuracy; transfer learning after compression improved results; 87 ms response time suitable for low- resource devices.
Ahmed, 2021	DCPLD-CNN	Develop CNN-based model for detecting cotton diseases (bacterial blight, rolling-leaf disorder)	Achieved 98.77% accuracy using transfer learning & augmentation; reduces need for manual diagnosis.
Tiwari et al., 2021	DenseNet	Automate plant disease detection using dense CNN to overcome limitations of traditional methods	Achieved 99.58% accuracy, high specificity (99.97%), real-time processing; effective for automated identification
Sharma et al., 2020	F-CNN & S-CNN	Improve disease detection *via* segmentation for robust CNN-based diagnosis	S-CNN (segmented images) achieved 98.6% accuracy, reduced misclassification, more robust than F-CNN
Chen et al., 2020	INC-VGGN	Enhance plant disease detection using transfer learning (VGGNet + Inception) with fewer labeled datasets	Achieved 91.83% validation accuracy; efficient for real-time monitoring & computer-aided diagnosis
Geetharamani and Pandian, 2019	Deep CNN	Develop a 9-layer CNN for accurate plant leaf disease classification	Achieved 96.46% accuracy; outperformed traditional ML models using data augmentation
Jiang et al., 2019	INAR-SSD	Develop real-time apple leaf disease detection using enhanced CNN (Inception + Rainbow concatenation)	Achieved 78.80% mAP & 23.13 FPS for five apple leaf diseases; improved speed & precision for on-field use
Barbedo, 2019	GoogLeNet	Improve plant disease detection by focusing on leaf lesions using CNNs and a large dataset (46,409 images, 79 diseases, 14 species)	Achieved 12% accuracy improvement; emphasized dataset diversity, collaboration, and citizen science for better real-world
Zhang et al., 2019	TCCNN	Improve vegetable leaf disease diagnosis by addressing segmentation & background issues using RGB channels	Achieved higher accuracy without manual segmentation; robust against lighting & background interference
Barbedo, 2018	LeNet, AlexNet, GoogLeNet, VGG	Examine factors influencing DL performance in plant disease detection and adoption challenges	Identified intrinsic/extrinsic factors (dataset diversity, misclassification, covariate shift) impacting accuracy; emphasized practical adoption issues
Lu et al., 2017	Deep CNN	Develop automated rice disease diagnosis using CNNs with standard digital cameras for early detection	Outperformed conventional methods with 95.48% accuracy, faster training, and user-friendly application
Dyrmann et al., 2016	Deep CNN	Use CNNs for precise weed and crop species recognition to improve site-specific weed control	Classified 22 weed & crop species with 86.2% accuracy, outperforming earlier approaches

Rastogi et al. (2024) proposed a Convolutional Neural Network (CNN) which aims to develop a plant disease classifier for potato, tomato, and pepper bell plants to precisely identify diseases and provide prompt treatments for more robust crops. It draws attention to the necessity of more accurate techniques, cutting-edge tactics including transfer learning, machine learning integration, and resolving system limitations for increased performance and generalization in CNN-based plant disease identification. The paper shows how AI may increase crop yields, lower losses, and support sustainable agriculture in India by exhibiting the success of a CNN-based model with 86.21% accuracy on the Plant Village dataset. Despite improving CNN-based plant disease detection, the study has some drawbacks, including a narrow illness scope, limited generalization, real-world complexity, scaling problems, dataset dependency, and the need for additional research for wider applicability. To improve model performance and practical usability for farmers, further studies on plant disease detection with CNNs should focus on transfer learning, real-time detection, optimisation strategies, and advanced ML integration.

Zhou et al. (2024) proposed REM-ShuffleNetV2, an improved lightweight model based on ShuffleNetV2 that provides accurate crop leaf disease detection in challenging field circumstances. According to the results, REM- ShuffleNetV2 performs better in crop disease detection than models such as DenseNet121 and EfficientNet, obtaining superior accuracy (96.72%) and F1 score (96.62%). It also has improved efficiency, attention mechanisms, and robustness, making it a useful tool for raising agricultural productivity. The study outlines future to expand datasets, improve model efficiency, and enable real-time agricultural applications, while acknowledging limitations like the need for broader environmental evaluations, limited disease sample types, and deployment challenges on mobile devices and field robots. To increase agricultural disease detection's practicality and resilience in agriculture, future research will focus on expanding data collection, creating effective deep learning models, optimizing for mobile deployment, integrating into autonomous systems, and testing in a variety of environments.

The goal of Islam et al. (2023) is to lower the crop losses and boosting cotton production in areas like Bangladesh, the suggested deep learning-based cotton leaf disease identification method makes use of improved Transfer Learning models (VGG-16, VGG-19, Inception-V3, and Xception) to boost accuracy and facilitate a web-based intelligent system for real-time prediction of plant diseases. The study demonstrates that refined deep learning models—particularly Xception, which has an accuracy of 98.70%—effectively identify cotton leaf diseases, facilitate real-time predictions through a web-based application, and provide a scalable framework for crop disease diagnosis. Despite its high accuracy, the suggested cotton disease detection model has drawbacks, including class imbalance, poor flexibility, insufficient feature extraction, and the requirement for sophisticated image pre- processing techniques like segmentation, which highlight areas for further development. To increase agricultural productivity and sustainability, future research on cotton disease detection should address class imbalance, improve feature extraction, integrate advanced pre-processing, improve adaptability to novel diseases, integrate IoT for real-time monitoring, expand to other crops, and investigate hybrid deep learning approaches.

To enhance the accuracy and effectiveness of agricultural query classification, Zhang and Wang (2023) present BERT-BiGRU-CapsNet with Attention Pooling (BBGCAP), a unique approach for categorizing inquiries pertaining to crop diseases and pests using BERT, BiGRU, attention pooling, and CapsNet. The report emphasizes the need for integrated research to improve classification and apply deep learning in agricultural Q&A systems by highlighting research gaps in crop disease and pest classification, including fragmented knowledge, limited datasets, complex queries, and real-time processing demands. According to the results, the BBGCAP model yields better performance compared to existing conventional techniques in the classification of agricultural diseases and pests, with over 90% precision, recall, and F1 scores. Its efficient architecture and enhanced performance on larger datasets make it a useful tool for real-time agricultural Q&A systems. To recommend areas for future optimization, the document identifies constraints such as the inability to handle complex semantic information, the dependence.

on big datasets, the limited applicability outside of agriculture, and the difficulties in accessing fragmented knowledge. To increase the BBGCAP model's efficacy in agricultural Q&A systems and pest management tactics, the paper makes several recommendations for improvements, such as optimization, data augmentation, knowledge graph integration, fine-tuning BERT for agriculture, ensemble learning, and adding multi-modal data.

Borhani et al. (2022) aims to design a lightweight deep learning model based on Vision Transformer (ViT) for real-time classification of plant diseases, enabling farmers to act early and preserve agricultural productivity which provides visual information. The report identifies research gaps in automated plant disease classification. These include the need to advance multi-label classification and object localization techniques, optimize real-time prediction speed, balance lightweight models with transfer learning, address imbalanced datasets, and adapt domains for small datasets. The report highlights the balance between prediction accuracy and speed, input image resolution, and real-world usability to support more effective crop management. It also shows that the Vision Transformer (ViT) Model 4, which has transformer blocks, performs better than conventional CNNs in F1-score, recall, and precision with fewer parameters. The paper lists the drawbacks of the Vision Transformer-based method for classifying plant diseases, such as speed and accuracy trade-offs, computational demands on smaller datasets, difficulties with generalization, and problems with image quality, multi-label classification, and unbalanced data. It also identifies areas that require more study. To enhance the efficiency and resilience of agricultural automated plant disease identification systems, future research proposes combining object localization and multi-label classification networks with the collection of well-labeled datasets.

To improve accuracy and efficiency through pruning methods, transfer learning, and model compression, Zhu et al. (2022) intends to create an optimized cotton disease identification method utilizing deep convolutional neural networks (DCNN) for deployment on mobile and smart devices with low resources. The report identifies research gaps in the areas of evaluating transfer learning and model compression, balancing accuracy, and efficiency, addressing imbalanced datasets in disease classification, optimizing model deployment for resource-constrained devices, and investigating novel architectures for various agricultural scenarios. With an average response time of 87 ms, the study showed that pruned models are feasible for effective, precise disease identification on resource- constrained devices. It also discovered that pruning DenseNet40 for cotton disease identification achieved 97.23% accuracy, with transfer learning following model compression outperforming the reverse approach. Several challenges are associated with applying pruning techniques to deep learning models for cotton disease identification such as the generalisability of pruning and transfer learning, the variety of datasets, the specificity of pruning techniques that might not fully address accuracy in complex or uncommon disease cases, and difficulties deploying on devices with limited resources. Future research directions for cotton disease identification are suggested in the document. These include hybrid models, lightweight networks, sophisticated pruning algorithms, real-time learning, unsupervised learning, multi-modal data integration, and enhanced data augmentation to improve model accuracy, efficiency, and adaptability for agricultural applications based on smart devices.

The goal of Ahmed (2021) is to create the Diseased Cotton Plant Leaf Detection Convolutional Neural Network (DCPLD-CNN) model for the autonomous identification of cotton plant diseases, including bacterial blight and a new rolling-leaf disorder, by making use of deep learning and convolutional neural networks. As a result, manual expert evaluations will no longer be necessary, allowing for rapid and accurate diagnosis. The paper highlights several important research gaps in the recognition of cotton plant disorder, such as the dearth of data, the requirement for region-based segmentation, the dependence on conventional image processing, and the need for improvements in methodology, data collection, and automatic feature extraction. The DCPLD-CNN model, which has a 98.77% accuracy rate in cotton disease diagnosis, is introduced in this work. This model demonstrates how pre-trained models, data augmentation, and transfer learning can enhance disease diagnosis and support crop management. This research points out shortcomings in generalization, model robustness, expert dependency, and data scarcity, indicating the need for larger datasets and better real-world application for more thorough disease detection in various agricultural contexts. To enhance the accuracy, usability, and real-world application of cotton plant disease recognition systems, future research directions include identifying disease severity, growing datasets, applying region-based segmentation, integrating cross-disciplinary insights, creating user-friendly mobile apps, and adding feedback mechanisms.

Tiwari et al. (2021) proposed a system based on deep learning that uses a Dense Convolutional Neural Network (DenseNet) to automate the identification and classification of plant illnesses, the research aims to address problems like as the inexperience and high expense of traditional diagnostic processes. The report identifies.

research gaps in plant disease identification, including the shortcomings of traditional methods., the requirement for a variety of datasets, integration with autonomous systems for monitoring in real time, and the necessity of comparative analysis of deep learning models in different agricultural environments to verify their effectiveness. In comparison to previous models, the work shows that a dense CNN is effective for automated plant disease identification, achieving 99.58% accuracy, real-time processing, and enhanced metrics (e.g., specificity: 99.97%) on a variety of datasets. Furthermore, there is potential for its integration with camera-based systems for continuous monitoring and disease management. Although the suggested dense CNN for plant disease identification exhibits remarkable speed and accuracy, it has drawbacks, such as the requirement for a more varied dataset, validation in real-world scenarios, and more extensive testing on unseen photos and other crops to verify its generalizability. The study makes recommendations for future research on growing datasets of plant leaf images, creating sophisticated AI systems using transfer learning and EfficientNet architectures, and integrating hyperspectral imaging to improve the effectiveness, precision, and robustness of systems for identifying plant species and detecting diseases.

To improve automated disease detection for effective crop management and food security, the Sharma et al. (2020) uses image segmentation to improve CNN models for identifying plant diseases. It demonstrates that a model trained on segmented images (S-CNN) achieves higher accuracy than one trained on full leaf images (F-CNN). By training CNNs, the study closes a gap in traditional deep learning models for diagnosing plant diseases with segmented photos targeted at symptomatic regions. For real world agricultural applications, this enhances accuracy and robustness while emphasising the significance of complex data preparation and segmentation. With the segmented CNN (S-CNN) attaining 98.6% accuracy, decreased misclassification, increased robustness, and useful implications for efficient automated disease detection in agriculture, the study shows that image segmentation greatly enhances CNN performance for tomato plant disease detection. The S-CNN model for identifying plant diseases has drawbacks, according to the paper. These include difficulties with overlapping symptoms, a dependence on segmentation quality, a lack of representation in the dataset, and the need for more extensive datasets or improved data augmentation to increase accuracy and consistency. By increasing the precision, robustness, and generalization of plant disease detection systems, more research could concentrate on advanced image segmentation, augmented data sourcing, GANs, multi-task learning, environmental feature integration, real-time processing, federated learning, and transfer learning refinements to enhance agricultural practices and food security.

Chen et al. (2020) proposed INC-VGGN deep transfer learning architecture aims to enhance accuracy and decrease the need for expensive labeled datasets and processing resources for plant disease detection using pre- trained CNN models such as VGGNet and Inception. To improve precision, decrease computing complexity, and increase performance in a variety of agricultural situations, the study highlights shortcomings in conventional plant disease identification techniques and suggests utilizing deep transfer learning with the modified CNN architecture INC-VGGN. Using VGGNet modifications to improve performance, the paper shows that the INC- VGGN deep transfer learning architecture is effective for plant disease identification, achieving 91.83% validation accuracy. It also suggests future applications in computer-aided diagnosis for plant health management and real- time monitoring on mobile devices. The study identifies the drawbacks of conventional plant disease detection techniques, such as their dependence on expert judgment, subjective feature selection, lack of diversity in data, difficulties in gathering sizable labeled datasets, and susceptibility to background fluctuations, which make it more difficult to create efficient automated detection systems for agricultural applications. Real-time mobile deployment, application to computer-aided diagnosis, improved image processing, investigating advanced neural network architectures, employing larger datasets, incorporating environmental context, and investigating explainable AI techniques for improved model interpretability and trust are some of the future directions for improving plant disease identification that are suggested in the document.

Geetharamani and Pandian (2019) aims to create a new type of deep convolutional neural network (Deep CNN) model that can reliably and efficiently classify plant leaves as either healthy or ill, outperforming traditional machine learning techniques. The objective of the paper is to increase plant disease identification accuracy by deep transfer learning using CNN models that have already been trained, such as VGGNet and Inception. This will decrease the need for expensive labelled datasets and processing power. This would help with efficient agricultural management. With the help of data augmentation and reliable metrics, the study shows that the nine- layer Deep CNN model for plant leaf disease identification outperforms conventional models with an accuracy of 96.46%. It also makes recommendations for future research, including the expansion of datasets, application to other plant parts, and investigation of unsupervised learning for precision agriculture. Potential difficulties are deduced from the study, such as the use of augmented images with little diversity, the emphasis on disease identification based on leaves, transfer learning constraints, overfitting risks despite dropout, and the requirement for additional research to increase dataset size, class diversity, and applicability to more extensive plant disease scenarios. Future research directions are outlined in the publication, and they include growing the dataset, optimizing the model for better performance, applying the model to different plant components, moving toward the practical detection of plant diseases, and investigating unlabeled training techniques to improve generalization.

Jiang et al. (2019) aim is to develop a revolutionary deep learning method namely INAR-SSD (SSD with Inception module and Rainbow concatenation) for the real-time identification of common apple leaf diseases using enhanced convolutional neural networks. By improving the speed and precision of apple leaf disease detection, this will benefit the Apple Companies. With an emphasis on enhancing accuracy, speed, and real-time application, the study fills a research gap in conventional methodologies and object recognition algorithms for apple leaf disease diagnosis. This results in the creation of an improved CNN strategy for useful on-field detection. The INAR-SSD deep learning model, which enhances existing methods and promotes sustainable apple production, is presented in the paper. Using cutting-edge technologies such as Rainbow concatenation and GoogLeNet Inception, it can identify five apple leaf diseases in real time with 78.80% mAP and 23.13 FPS. The study highlights the need for improvements to increase the model's robustness and practicality by identifying issues such the inability to differentiate between comparable diseases, vulnerability to environmental influences, possible overfitting, and a trade-off between speed and accuracy. Future directions for improving the INAR-SSD model are outlined in the document. These include the utilization of GANs for advanced data augmentation, the integration of multi-modal sensor data, transfer learning, advanced feature visualization, real-world deployment with feedback loops, and the investigation of ensemble learning for increased robustness and adaptability.

Barbedo (2018) proposed GoogLeNet by concentrating on individual leaf lesions using CNNs, the study seeks to improve plant disease detection. To increase training data diversity and classification accuracy, a comprehensive dataset of 46,409 photos from 79 diseases across 14 plant species will be created. The study draws attention to the deficiency of small, homogeneous datasets for plant disease diagnosis, highlighting the necessity for more varied data and recommending citizen science and collaboration to increase dataset variety and boost the efficacy of deep learning models. By concentrating on certain lesions and using a diverse dataset of 46,409 images, the study shows that deep learning, particularly CNNs, improves plant disease detection accuracy by 12%. It also highlights the importance of data diversity, collaboration, and citizen science in image annotation for improved real-world applicability. The study identifies several barriers to full automation and practical use of deep learning for the identification of plant illnesses, like sample imbalance, generalization issues, manual segmentation requirements, dataset representativeness, and the resources and time required for data collection. The study makes recommendations for future advancements in the identification of plant diseases by utilizing deep learning, like adaptable network designs, automated symptom segmentation, innovative data augmentation, citizen science for data gathering, and cooperation for a wider range of datasets.

To solve segmentation issues and background interference, Zhang et al. (2019) present a Three-Channel Convolutional Neural Network (TCCNN) model for vegetable leaf disease diagnosis. To improve classification accuracy, this model takes advantage of RGB picture colour information. This study introduces a three-channel convolutional neural network (TCCNN) for the recognition of vegetable leaf disease. By using RGB colour components to improve accuracy without manual segmentation, the network overcomes drawbacks like poor segmentation, the requirement for extensive preprocessing, and sensitivity to lighting changes. This work represents a breakthrough in agricultural disease surveillance by introducing a Three-Channel Convolutional Neural Network (TCCNN) for vegetable leaf disease detection. This network decreases preprocessing, increases accuracy, and strengthens resilience against real-world picture variability. The research identifies opportunities for additional validation by highlighting the drawbacks of conventional techniques, such as complicated backdrops and manual segmentation, which may also have an impact on the TCCNN model for vegetable leaf disease detection. To enhance the recognition of vegetable leaf diseases, the paper highlights the necessity of developments in image segmentation and feature extraction and recommends investigating methods such as data augmentation, transfer learning, multimodal data, attention mechanisms, ensemble learning, and real-time monitoring.

To reduce the need for specialized knowledge and provide farmers with a simple tool for early detection using standard digital cameras, Lu et al. (2017) suggests building an automated rice disease diagnosis method that uses Deep convolutional neural network (CNN) to improve diagnostic speed and precision. To improve the effectiveness and precision of diagnosis, the research proposes deep CNNs as a remedy for the inadequacy of conventional expert knowledge-based rice disease identification techniques. The study demonstrates how well a CNN-based model for rice disease diagnosis works, surpassing conventional techniques in automated detection with 95.48% accuracy, quicker training, and an intuitive application. To improve the precision of automated rice disease detection, the research identifies several obstacles to CNN design optimization, such as the requirement for sizable, high-quality datasets and the necessity to adjust parameters. To improve precision and early disease diagnosis, the paper describes future directions for automated rice disease identification, with a particular emphasis on investigating other deep architectures, training techniques, expanding datasets, and fine-tuning CNN parameters.

By employing Deep convolutional neural network (CNN) for precise plant species recognition, Dyrmann et al. (2016) seeks to improve weed management in agriculture by enabling site-specific weed control and lowering the use of herbicides to increase sustainability and yield protection. To overcome the constraints of manual feature extraction and improve site-specific weed control for the best herbicide use, the study employs deep convolutional neural networks (CNNs) to categorize 22 weed and crop species. The CNN model presented in this work outperformed earlier research and showed promise for site-specific weed management in agriculture, classifying 22 weed and crop species with an accuracy of 86.2%. To create a more reliable and adaptable weed and crop classification system, the report identifies shortcomings in earlier research, including restricted species recognition, dependency on pre-processing, class biases, and data unpredictability. To increase resilience, accuracy, and efficiency in agricultural applications, the research makes recommendations for future advancements in the categorization of weed and crop species. These include improved data augmentation, transfer learning, multispectral imaging, hybrid techniques, and real-time processing.

## 3 Proposed methodology

**Figure 1:**
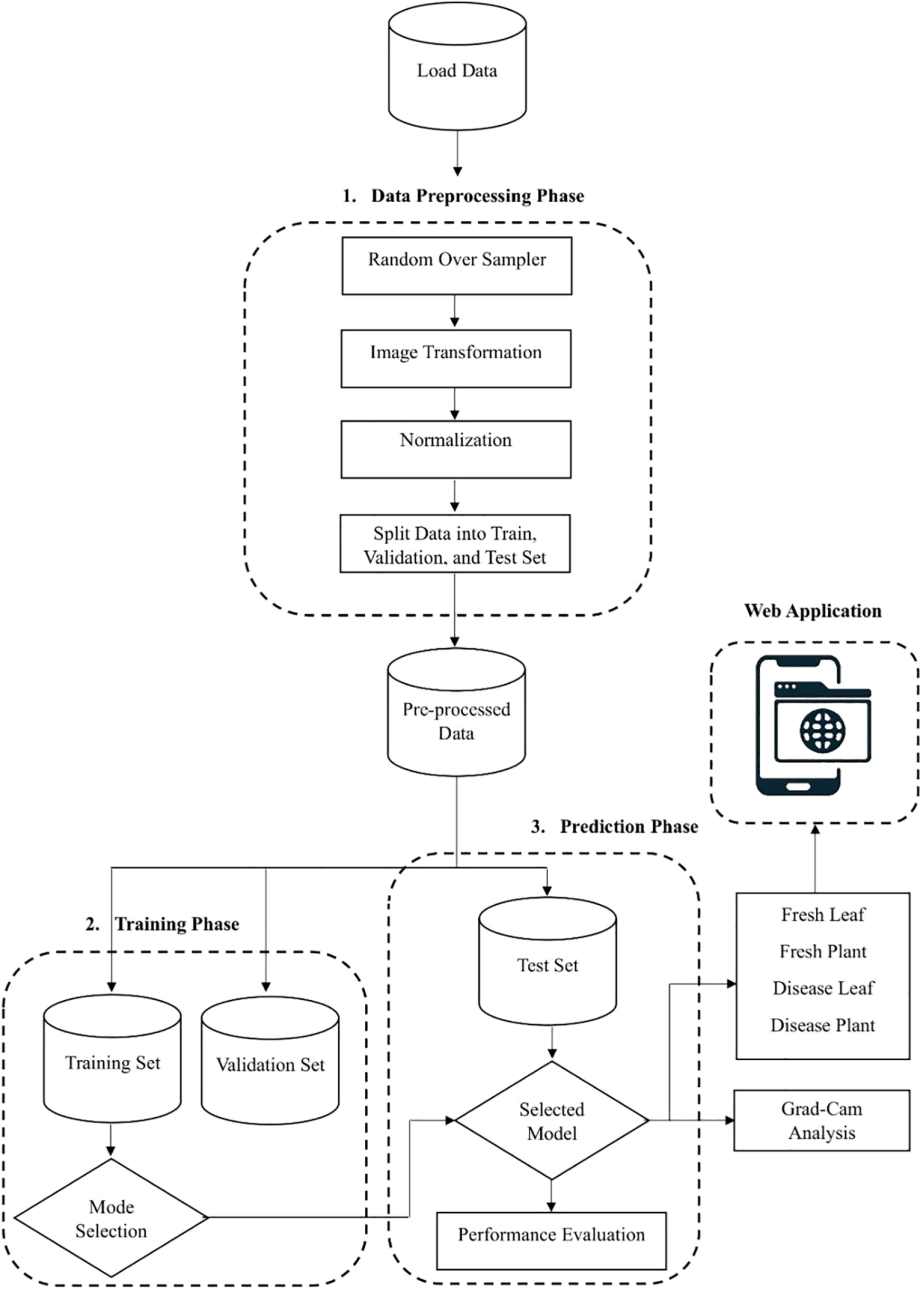
Block diagram of proposed methodology.

**Figure 1** depicts the complete pipeline of the suggested model. Three primary steps make up the approach: preparing the data, training the model with the training dataset, and making predictions with the test dataset. Each step's specifics are given in the following subsections.

### 3.1 Data preprocessing step

- Normalization: Image normalization is a preprocessing strategy that standardizes the size and pixel range of all images in the dataset. This procedure is essential to improving the model's generalizability and facilitating faster convergence during the training stage.
- Image Transformation: The images are resized to 224×224 pixels to ensure uniform input size and smooth compatibility with the neural network.
- Random Oversampling: Random oversampling helps correct class distribution issues by expanding the dataset with repeated samples from the less frequent classes. To ensure a more balanced dataset, it randomly duplicates existing samples from the minority class. This method reduces biases toward majority classes which enhances the models' performance. However, because repeated samples do not contribute new information, it can result in overfitting.

- Splitting of Dataset: To support learning and performance assessment, the data is divided into segments for testing, validation, and training.
- a) Training Set: Most of the dataset is used to make the training set and this training set is used to fine-tune the model by updating its parameters through repeated learning cycles.
- b) Validation Set: Using the validation set, the model's performance is evaluated and its hyperparameters are adjusted. Because it permits evaluation on data that was not visible at the time of training, it is essential in avoiding overfitting.
- a) Test Set: Test set is used only when model training is complete the test set is a separate dataset. It evaluates the final performance and generalizability of the trained model to fresh data.

- Image Augmentation: Image augmentation is the modified version of the existing data which helps to increase the model performance and generalization. To improve both the volume and variety of training data, data augmentation techniques are applied. It plays a crucial role in improving the robustness and minimizing the risk of overfitting. Data augmentation is mainly used when we are leading with an unbalanced and limited dataset. Various transformation techniques like rotating, flipping, zooming, cropping, or adjusting brightness and contrast are applied to our existing images, aiming to prevent overfitting, improve model robustness, and allow deep learning models to generalize better when exposed to new or unseen data. Translation technique helps to learn the model of various positional contexts of the cotton leaves by shifting the images horizontally and vertically by 10% of their dimension. We employed horizontal flipping to generate the mirror images of the original images, and this technique provides a border spectrum of visual patterns and also helps the model to generalize across naturally occurring variations by strengthening the dataset's diversity.
- Web application Development: We have developed a web application using a Python framework, namely Flask, for deployment and runs DL based applications. This web application is capable of identifying cotton disease leaves and plants from healthy leaves and plants based on real-time data.
- Grad Cam Analysis: We used the Grad Cam analysis method, which plays a significant role by generating the heatmaps that indicate the disease region of the cotton plant and leaves that contribute most of the model’s predictions. It enhances the trust of plant pathologists by transforming the abstract neural network output into a visual explanation and which bridges the gap between AI and human understanding and makes the diagnosis process more transparent and reliable.

### 3.2 Model training phase

Step I: Define the Model:

Using pretrained backbone with custom layers involves leveraging a pre-trained deep learning model (e.g., VGG16, VGG19, InceptionV3, Xception, MobileNet), as a feature extractor or base for a new model. These pretrained models offer reliable feature representations because they were trained on huge datasets like ImageNet. The model is then customized for a particular task by adding custom layers:

- Reshape: Modifies the tensor's dimensions to fit the architecture that is desired.
- MultiHeadAttention: Frequently utilized in transformer topologies, this feature records relationships between various input components. MultiHeadAttention enables the model to focus on multiple regions of the cotton leaf images simultaneously. Multi Heda attention allowing to capture the contextual information from various spatial locations. By applying attention across multiple heads, the model can easily identify the different features and that improve the ability to detect the subtle patterns hat may be indicative of disease.
- Dropout: During training, neurons are randomly dropped to lessen overfitting.
- Regularization layers: By penalizing excessively complex models, they help avoid overfitting.

This method blends task-specific modifications with the effectiveness of transfer learning.

1. Pretrained Model: By leveraging previously learnt generic characteristics (such as edges and textures), models like MobileNet or VGG16 that have been pretrained on sizable datasets like ImageNet can speed up training. Through fine-tuning, this method enhances model performance on certain tasks while lowering the computational load and requiring a big dataset.
2. Unfreeze Specific Layers: To maintain generic properties like edges and textures, previous layers of a pretrained model are usually frozen in transfer learning. To adapt the model to a new task, deeper layers that capture more task-specific patterns can be unfrozen and adjusted, allowing for effective learning without erasing fundamental features.
3. Custom Layers: Add task-specific layers to a pretrained backbone to improve it:
  • Reshape: Transforms 2D features into attention-mechanism sequences.
  • MultiHead Attention: Improves feature extraction by concentrating on key areas.
  • Dropout: During training, neurons are randomly deactivated to prevent overfitting.
  • Regularization (L2): By penalizing big weights, it prevents overfitting.
  • Dense Layers: Incorporate fully connected layers according to the task's output dimensions.

4. Learning Rate Scheduler: Modifies the learning rate to improve convergence during training.

- Warm-Up: To stabilize the model early on, it starts with a low learning rate.
- Plateau: Maintains a steady learning rate throughout training phases.
- Decay: Adjusts the model's weights by gradually lowering the learning rate near the end.

5. Optimizer Configuration: Use an optimizer like Adam, which adapts the learning rate for each parameter, along with gradient clipping to prevent unstable updates by limiting large gradients, ensuring stable training and improved model convergence.

Step II: Compile the Model.

Setting up the model for training entails choosing the metrics (like accuracy) to assess performance, the optimizer (like Adam) to update weights, and the loss function (like sparse_categorical_crossentropy) to measure prediction error. Learning rate scheduling is employed to dynamically modify the learning rate during training, ensuring stable updates and better convergence of the model.

Step III: Train the Model.

Once the model is compiled, the next step is to train it using the augmented data while incorporating monitoring mechanisms to ensure optimal performance. Each step in this procedure operates as follows:

1. Feed Augmented Data: To expand the training dataset, the original images are subjected to data augmentation techniques. These include transformations like rotation, flipping, zooming, and color adjustments. Such variations help the model learn from diverse patterns, enhancing its ability to generalize and perform well under real-world conditions.

We expose the model to a wider variety of changes by feeding it enhanced data which helps it acquire more robust features and avoids overfitting and it is the process by which a model memorizes patterns.

2. Monitor Metrics:

- The model's learning progress and generalization strength are assessed throughout the training phase by monitoring key performance metrics like accuracy and loss for both training and validation.
- Validation Loss against Training Loss: Tracking the loss values during training helps to identify overfitting—an issue where the model excels on training data and does not work well with validation data. Instead of learning patterns that generalize to new inputs, the model has likely memorized the training data when the training loss is much smaller than the validation loss.

3. Callbacks:

- Early Stopping: To reduce the chance of overfitting and save computational effort, the callback mechanism is made to terminate training early when no additional reduction in validation loss is seen.
- Learning Rate Adjustment: During training, this callback adjusts the learning rate. The learning rate is lowered to enable more precise weight adjustments which aid in the model's better convergence, if the validation loss stagnates, that shows no progress after a predetermined number of epochs.

### 3.3 Prediction phase

The last stage, the Prediction Phase, tests the model's capacity to process fresh, untested data. This phase is critical because it gives a clear measure of how well the model generalizes to real-world situations.

### 3.4 Cotton net MHA model

The model architecture is expertly designed and it incorporates a sequence of convolutional and pooling layers, culminating in a cutting-edge Multi-Head Attention mechanism that effectively processes feature maps before the flattening stage. Initially, the model employs three effective convolutional layers, using 32, 64, and 128 filters in each layer. A MaxPooling2D layer, which employs a pool size of (2, 2), comes after each layer. This improves the model's ability to recognize and interpret key characteristics. To prevent overfitting and maintain strong performance, we incorporate the effective L2 regularization technique. This advanced architecture is designed to yield outstanding results.

Following the convolutional layers, the feature map undergoes normalization through a LayerNormalization layer, setting a solid foundation for the next step. The MultiHeadAttention mechanism is then employed, allowing for the capture of intricate dependencies within the feature maps. This advanced attention mechanism leverages four attention heads, each with a key dimension of 16, ensuring a robust analysis of the data that enhances overall model performance.

To effectively reduce the spatial dimensions, we integrate a GlobalAveragePooling2D layer into the model architecture, which transforms the output into a concise vector that aligns with the quantity of channels. This is succeeded by a robust design of two dense layers featuring 128 and 512 units, both leveraging the powerful ReLU activation function. To enhance the model's resilience and combat overfitting, we introduce a GaussianNoise layer that adds a touch of random noise. After the second dense layer, a dropout layer with a 0.2 rate is added to enhance the model's generalization. Lastly, we finish with a dense output layer that has four units and uses a softmax activation function to precisely classify the input into one of four classes.

## 4 Classification models

The following Deep learning models are used for performance analysis:

### 4.1 VGG16

Image classification, image recognition, and object identification are the tasks handled by the VGG16 convolutional neural network. The network's 16 layers of artificial neurons analysed image data gradually to improve prediction accuracy. Rather than employing several hyperparameters, with max pools of 2x2 filter and stride 2 and 3x3 filter and stride 1, VGG16 use convolution layers. The entire arrangement makes use of the convolution and max pool layer architecture. The result comprises a softmax for output and two fully connected layers. The final product consists of two completely connected layers and an output softmax. The time required for model training and optimization is reduced by the availability of VGG16 as a pre-trained neural network and its composition and quantity of layers. Furthermore, the number of layers and its structure provide extremely precise image categorization findings (Bagaskara and Suryanegara, 2021).

### 4.2 VGG19

There are 19 weight layers in this relatively deep convolutional neural network which has 16 convolutional layers and 3 fully connected layers. Its straightforward architecture and repetition make it easy to use and comprehend. Non-linearity is added after each convolution by the function that initiates ReLU and to preserve spatial resolution, convolutional layers employ 3x3 filters with padding and stride of 1. Max pooling layers can be used to effectively minimize spatial dimensions. Their filter is two-by-two, and they are two-steppers. Three fully connected classification layers make up the network and the final softmax layer produces the final class probabilities (Bagaskara and Suryanegara, 2021).

### 4.3 Inception V3

In 2015, Google unveiled Inception V3, a convolutional neural network design. To improve performance on image categorization tasks this improved version of the original Inception model uses fewer parameters. The Inception V3 architecture uses a "deep stem" of convolutional layers to take characteristics out of the input imeage, followed by several inception modules to record data at various sizes. Inception modules use average and maximum pooling to extract features and 1x1, 3x3, and 5x5 convolutional filters. To regularizing the model at the network's end and preventing overfitting, the model also includes dropout, batch normalization, and a global average pooling layers. A few image classification tests, such as the ImageNet Large Scale Visual Recognition Challenge (ILSVRC), have shown that Inception V3 performs exceptionally well (Hassan et al., 2021; Too et al., 2018).

### 4.4 Xception

Francois Chollet at Google created the Deep Learning model known as Xception or Extreme Inception by further refining and maintaining the Inception architecture. This deep convolutional neural network's architecture incorporates depth wise separable convolutions. Google defined Inception modules as a stage between the depth wise separable convolution operation and standard convolution which follows a pointwise convolution after a depth wise one in convolutional neural networks (Chollet, 2017). Depth wise Separable Convolution Resembling ResNet's convolution block shortcuts, this architecture is based on two essential elements.

The Xception model substitutes depth wise separable convolution layers which add up to 36 layers for the inception modules used in the inception architecture. The Xception model outperforms the Inception V3 model by a small margin on the ImageNet dataset while it outperforms the latter by a huge margin on larger datasets with 350 million images.

### 4.5 MobileNet

A powerful yet lightweight feature extraction architecture is MobileNet that was open-sourced by Google (Howard et al., 2017). It features minimal latency, cheap computing cost, great accuracy and smaller neural networks. When depth wise separable convolutions are used, the number of parameters is substantially reduced in comparison to previous networks that used standard convolutions and had the same net depth. The resultant deep neural networks are lightweight. This design makes it possible for convolutions to be separated based on depth. To produce a depth wise separable convolution, two procedures are utilized.

1. Depthwise convolution.
2. Pointwise convolution.

MobileNet can serve as the foundation for segmentation, classification, detection, and embeddings (Linkon et al., 2021).

## 5 Dataset and implementation details

### 5.1 Description of dataset

**Figure 2:**
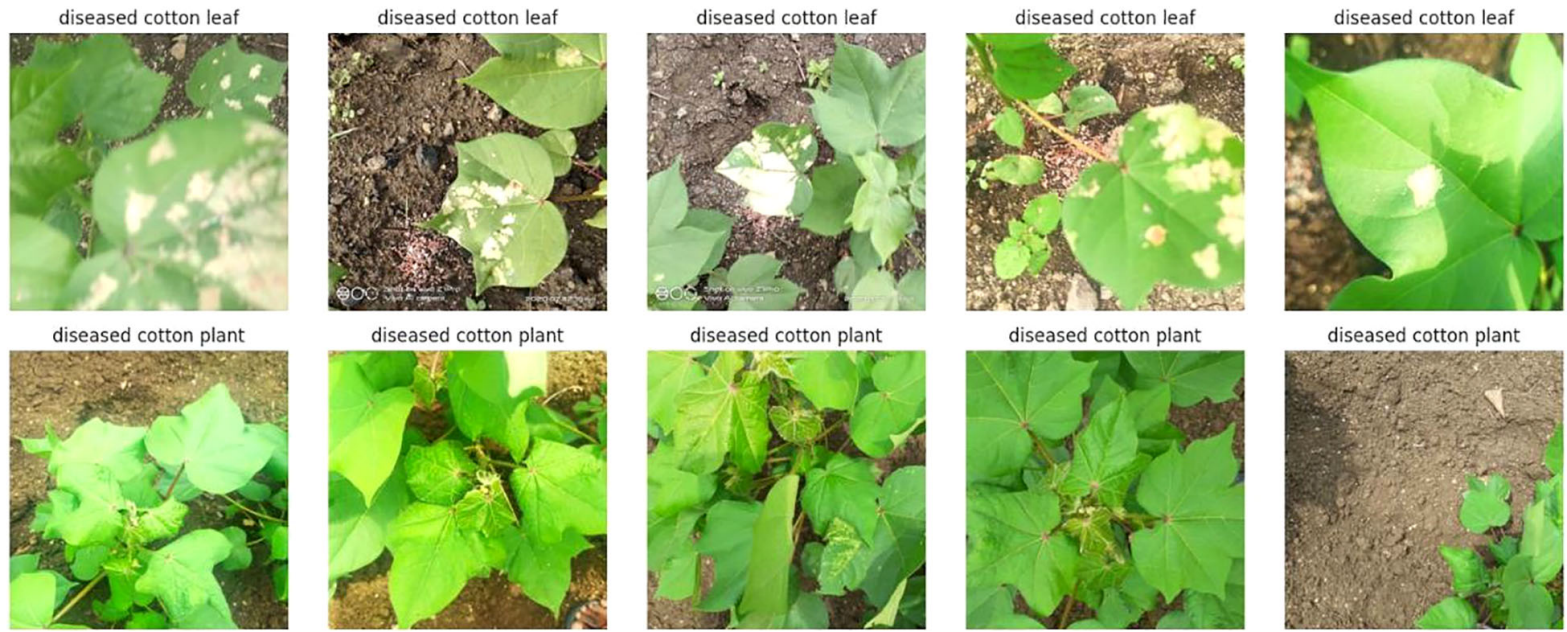
Sample dataset of cotton plant disease.

**Figure 3:**
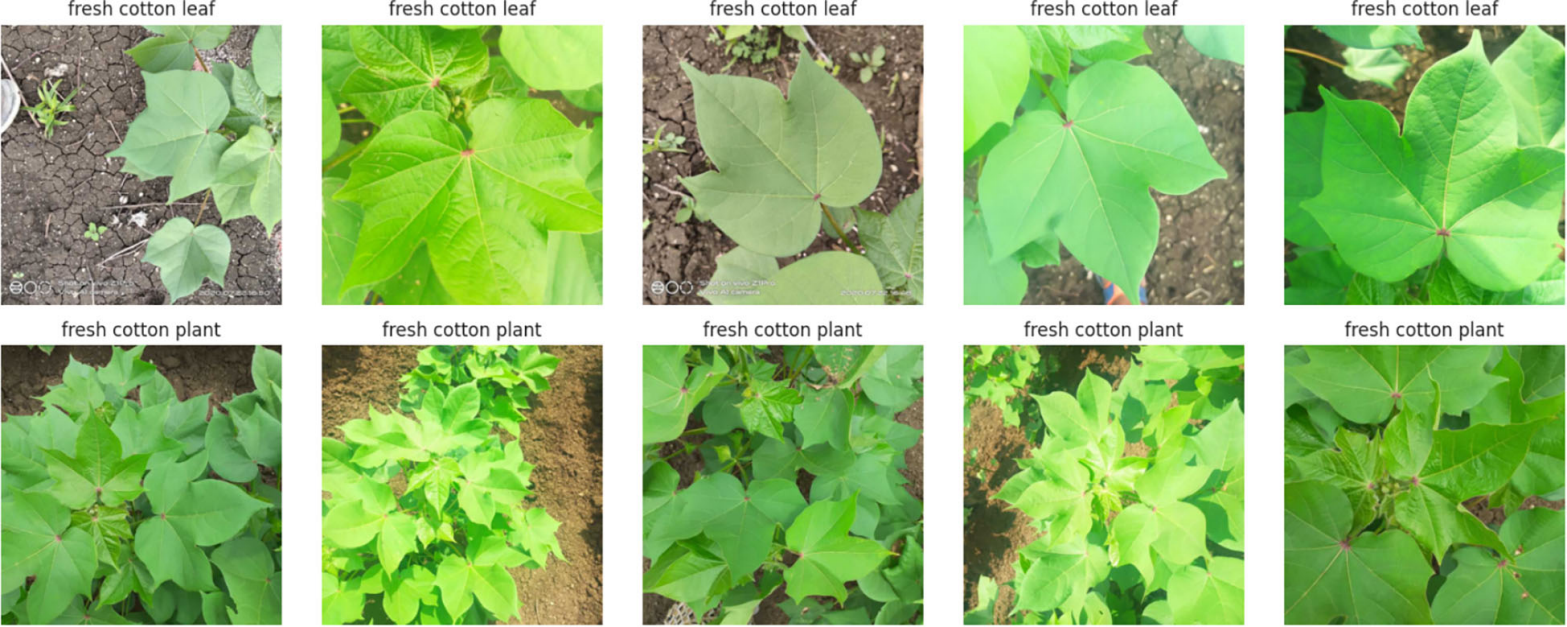
Sample dataset of fresh cotton plant.

We have collected a freely available dataset from Akash Zade. The dataset contains 2293 total images. The dataset has been classified into three categories (i)Training, (ii) Testing, and (iii) Validation as shown in **Figure 2** and **Figure 3**. Each category has 4 classes: (a) Disease cotton leaf, (b) Disease cotton plant, (c) Fresh cotton leaf, and (d) Fresh cotton plant. The training set contains 1951 images, 324 images for validation, and 18 images for testing. After applying augmentation techniques, the dataset size was increased to 19,520 images, ensuring a more diverse and balanced representation.

Cotton is susceptible to diseases that can damage its leaves and overall health, ultimately reducing its yield and quality. The spectrum of diseases includes.

- Cotton Leaf Curl Disease: A virus spread by whiteflies causes the newest leaves to become smaller, thicker, glossy, crinkled, and curl upward.
- Bacterial Blight: Small, angular, water-soaked spots on leaves that turn brown or black.
- Powdery mildew - The upper leaf surfaces get distorted and yellowed due to a white, powdery fungal growth.
- Target Spot: Brown, round, concentric-ringed leaf spots that frequently have a yellow halo around them.
- Aphids: These insects cause young leaves to curl and distort, and they are frequently covered in sticky honeydew, which can cause black sooty fungal growth.

The dataset used in this work is downloaded from Akash Zade (Data Scientist) which is openly accessible and can be found at: https://drive.google.com/drive/folders/1vdr9CC9ChYVW2iXp6PlfyMOGD-4Um1ue.

**Figure 4:**
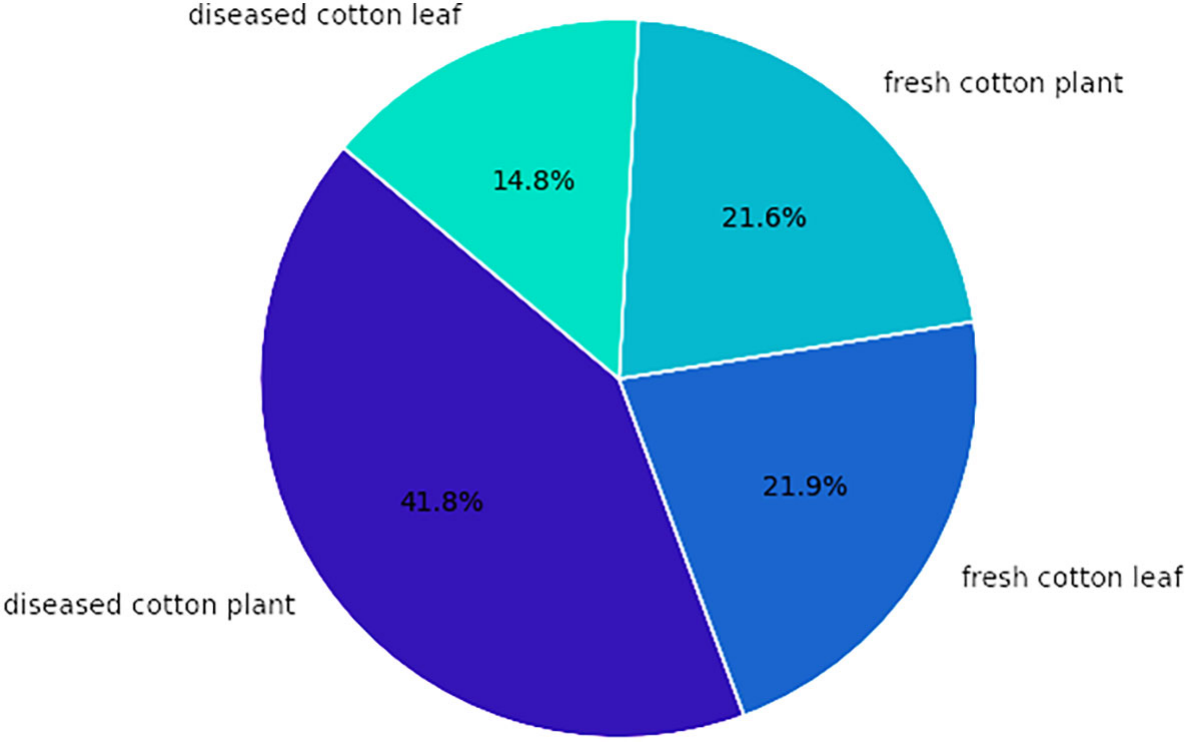
Distribution of cotton plants.

The dataset comprises four categories of cotton plants with their distribution illustrated in **Figure 4**. The diseased cotton leaf category accounts for 14.8% of the images, while diseased cotton plants make up 41.8%. Fresh cotton leaves constitute 21.9% of the dataset, and fresh cotton plants represent 21.6% of the images.

**Figure 5:**
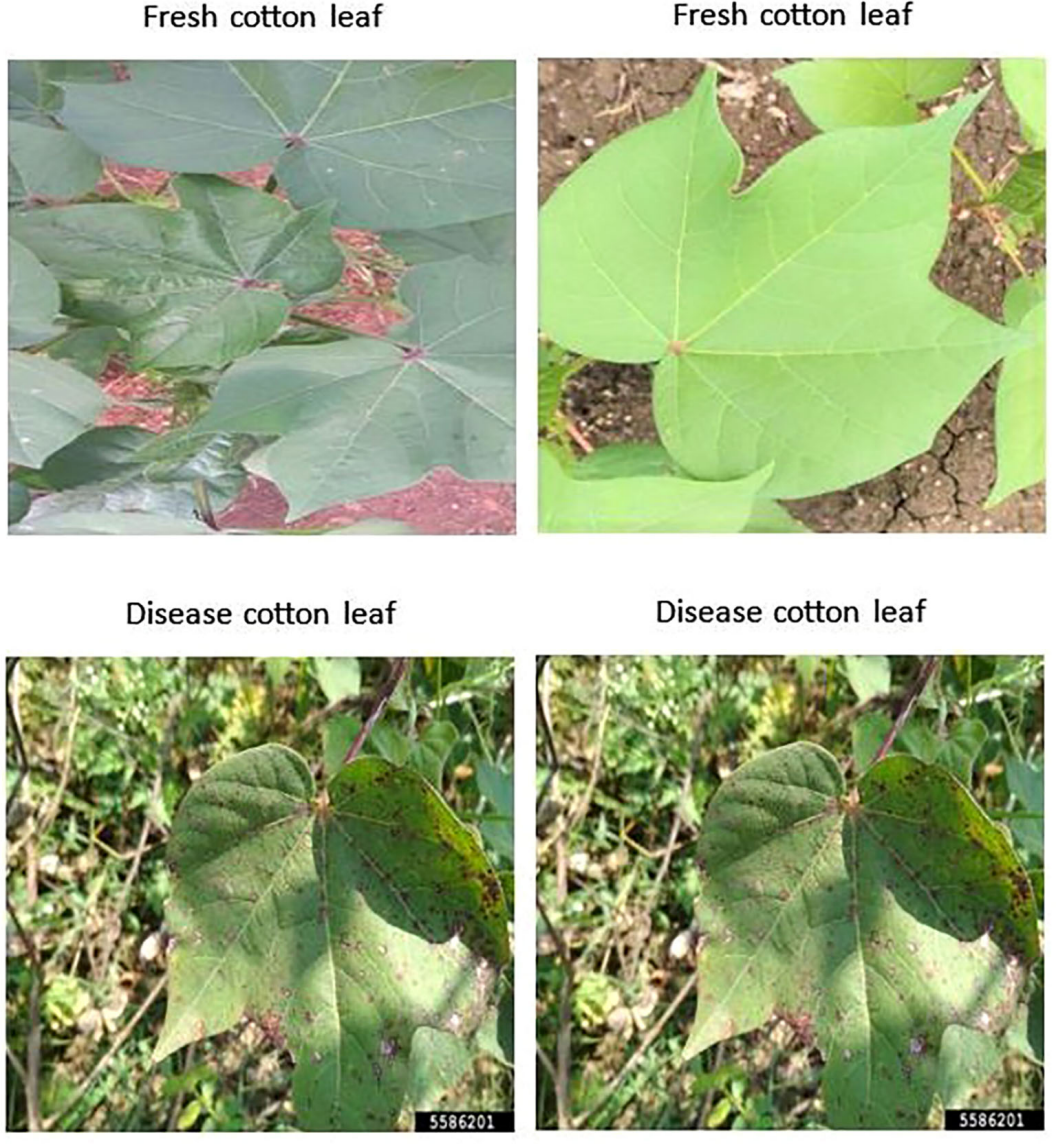
Serosh Karim sample dataset.

For additional testing, a dataset from Kaggle (Noon et al., 2021) was utilised, containing images of cotton plants and leaves exhibiting different diseases as shown in **Figure 5**. The dataset includes labelled samples, making it suitable for assessing the model’s accuracy and ensuring reliable performance across multiple disease categories. The dataset contains a total of 1710 images and includes four classes. Images of these dataset collected from real-world scenarios as well as the internet. The link to the dataset is given below: https://www.kaggle.com/datasets/seroshkarim/cotton-leaf-disease-dataset

### 5.2 Tools selection

The proposed model is implemented using Python 3.10.8. Keras Deep Learning Version 2.9.0 with TensorFlow support is used for model training. The proposed system uses version 2.10.1 of Tensor Flow and uses the graphical user interface; numerous trial setups are conducted to evaluate the performance. A GPU rather than a CPU is used for testing and training purposes. The proposed model will be implemented in the Jupyter notebook environment.

### 5.3 Implementation details

The following parameters are used for performance evaluation:

*Confusion matrix:* This table summarizes the performance of the classification model, which supports both binary and multiclass classification. Correct predictions are denoted by true positives (TP) and true negatives (TN), whereas false negatives (FN) and false positives (FP) represent missed detections and false alarms, respectively.

*Accuracy:* This metric assess how well a machine learning model predicts the correct outcome which is expressed in **Equations 1** and **2**

(1) $$Accuracy=\frac{Correct\;Predictions}{All\;predictions}$$

(2) $$Accuracy=\frac{TP+TN}{TP+TN+FP+FN}$$

Precision: Precision quantifies the proportion of positive predictions made by the model which is defined in **Equation 3**. In other words, precision evaluates the reliability of the model by indicating the likelihood that a predicted positive case is truly positive.

(3) $$Precision=\frac{TP}{TP+FP}$$

Recall: Out of all the actual positive samples in the dataset, the frequency with which a machine learning model properly detects positive instances—also known as true positives—is measured, which is given in **Equation 4**.

(4) $$Recall=\frac{TP}{TP+FN}$$

*F1 – score:* This metric is determined by using the harmonic mean of precision and recall. This approach merge precision and recall into a single evaluative parameter to maintain the effective balance between them.

The F1-score can be mathematically expressed as shown in **Equation 5**

(5) $$F1-Score=\frac{2*Precision*Recall}{Precision+Recall}$$

Support: Support denotes the number of instances of each class present in the dataset.

*Loss curve:* The loss curve, sometimes referred to as the training loss curve, shows how the performance of the model changes over time by computing the inaccuracy (or dissimilarity) between the model's expected and actual outputs. The loss indicates the degree to which the actual values deviate from the model's predictions.

*ROC - AUC Score:* Receiver Operating Characteristic Area Under the Curve is referred to as ROC AUC. It provides a single value that indicates the overall performance of the classifier across various classification thresholds. This is the area below the ROC curve. It summarizes the ability of the model to produce relative scores for distinguishing between different classes across all classification thresholds. An ROC AUC value of 0.5 indicates random guessing, while a value of 1 represents perfect classification performance. ROC AUC defines the overall quality of the model across different thresholds, providing a deeper understanding of its performance evaluation.

The **Table 2** presents a comparison of hyperparameters between existing models and the proposed model for a deep learning approach. The significance of each hyperparameter is given below:

**Table 2:** Hyperparameters used in deep learning model.

Hyperparameter	Value	
	Existing models	Proposed model
Learning rate	0.0001	0.0007
Batch size	16	32
Number of epochs	10	50
Optimizer	Adam	Adam
Activation	Relu	Relu
Dropout	0.3	0.2
Gaussian noise	0.25	0.2
Kernel size	3×3	3×3
Loss	Sparse_categorical_crossentropy	Sparse_categorical_crossentropy
Image size	224×224	224×224
Multihead attention	8	4
Early stopping patience	5	6

- Learning Rate: During training, the learning rate regulates how much the model modifies its parameters. Although a greater learning rate (0.0007 in the suggested model) implies faster updates and might result in faster convergence, there is a chance that the ideal solution will be overshot.
- Batch Size: The number of training data points processed prior to model updates is known as the batch size. Reliable gradient updates and more effective training could result from the suggested model's larger batch size (32).
- Number of Epochs: The model's frequency of running through the complete dataset is determined by the number of epochs. More epochs are covered by the suggested model to learn more intricate patterns (50 vs. 10), increasing the chance of overfitting.
- Optimizer: The optimization method Adam (Adaptive Moment Estimation) incorporates the advantages of momentum and RMSprop optimizers to enable efficient training.
- Activation Function: By adding non-linearity using the Rectified Linear Unit (RLU) activation function, the model may learn complex patterns.
- Dropout: To avoid overfitting, a portion of neurons are arbitrarily discarded during training as part of the dropout regularization strategy. The suggested model appears to strike a balance between regularization and learning ability with a marginally lower dropout rate (0.2).
- Gaussian Noise: To provide unpredictability and strengthen the model, Gaussian noise is employed as a regularization technique. Gaussian noise has marginally lower noise, the suggested model may rely more on learnt features than on chance variations.
- Kernel Size: In convolutional layers, the filter size is referred to as kernel size. CNNs often use a 3x3 kernel, which strikes a reasonable compromise between computational efficiency and collecting local information.
- Loss Function: When working with integer-labeled categorical data, this loss function is particularly useful for multi-class classification issues.
- Image Size: For deep learning models like CNNs, the input image size stays constant at 224 x 224 pixels, which is a common resolution.
- Multi-head Attention: In transformer-based models, multihead attention is a system that enables the simultaneous attention of various input components. By lowering the number of attention heads from eight to four, the suggested model may lower computational complexity without sacrificing performance.
- Early Stopping Patience: This refers to how many epochs without progress are required before training is stopped. A marginally greater patience value (6), the suggested model can train for longer periods of time before ceasing, perhaps leading to improved generalization.

The following observations were made while selecting the hyperparameters for the proposed mobility model:

- The suggested approach emphasizes faster learning and reliable updates by using a bigger batch size and a higher learning rate.
- A deeper training process is suggested by the increase in epochs.
- Confidence in model generalization is shown by lower Gaussian noise and dropout.
- More flexibility in training is possible with a little higher early stopping patience; fewer attention heads indicate an optimization of computing resources.

## 6 Result and discussion

In-depth research and data analysis are presented in this section, which serves as the core of the investigation. The previous sections provided an overview of the study's objectives and methodology; now, we focus on the key findings from our analysis. Using a range of performance indicators, we provide a clear and insightful review of the deep transfer learning models which we have implemented. Advanced optimization techniques, including learning rate (learning rate = 0.0001), early stopping (patience = 5 epochs), Gaussian noise (σ = 0.25), dropout (rate=0.30) and L2 regularization (λ = 0.0005), and multi-head attention (attention heads=10) have been systematically refined to enhance the efficiency of deep learning models in detecting and classifying cotton plant diseases.

The effectiveness of many deep learning classifiers in identifying four classes of cotton plant images—diseased cotton leaf, diseased cotton plant, fresh cotton leaf, and fresh cotton plant—is contrasted in **Table 3**. Metrics like Precision, Recall, F1 Score, Overall Accuracy, and Test Loss are used to evaluate the models. The analysis reveals that VGG16 reaches 94% total accuracy with a test loss of 0.1368, demonstrating strong performance across all categories. It records the highest F1 score (0.99) for diseased cotton leaf, while the lowest (0.90) is observed for fresh cotton plant. In contrast, VGG19 exhibits a lower overall accuracy of 89% and a higher test loss of 0.2874, with the lowest F1 score (0.91) for diseased cotton leaf, indicating challenges in classification.

**Table 3:** Execution of Applied models for cotton data set.

Classifiers	Class	Precision	Recall	F1 score	Overall accuracy	Test loss
VGG16	diseased cotton leaf	1.00	0.99	0.99	0.94	0.1368
	diseased cotton plant	0.93	0.94	0.93		
	fresh cotton leaf	0.95	0.94	0.94		
	fresh cotton plant	0.89	0.90	0.90		
VGG19	diseased cotton leaf	0.96	0.86	0.91	0.89	0.2874
	diseased cotton plant	0.93	0.86	0.90		
	fresh cotton leaf	0.83	0.95	0.89		
	fresh cotton plant	0.85	0.87	0.86		
InceptionV3	diseased cotton leaf	0.99	0.89	0.94	0.93	0.1524
	diseased cotton plant	0.97	0.93	0.95		
	fresh cotton leaf	0.85	1.00	0.92		
	fresh cotton plant	0.94	0.91	0.92		
Xception	diseased cotton leaf	0.96	0.99	0.98	0.96	0.1317
	diseased cotton plant	0.97	0.89	0.93		
	fresh cotton leaf	0.99	0.96	0.98		
	fresh cotton plant	0.91	0.99	0.95		
MobileNet	diseased cotton leaf	1.00	1.00	1.00	0.98	0.0686
	diseased cotton plant	1.00	0.99	0.99		
	fresh cotton leaf	0.94	1.00	0.97		
	fresh cotton plant	1.00	0.95	0.97		
CottonNet- MHA	diseased cotton leaf	1.00	1.00	1.00	0.99	0.0683
	diseased cotton plant	1.00	0.98	0.99		
	fresh cotton leaf	0.98	0.99	0.98		
	fresh cotton plant	1.00	1.00	1.00		

InceptionV3 shows an improved accuracy of 93% with a test loss of 0.1524. It excels in classifying fresh cotton leaf (Recall: 1.00) but faces minor difficulties in identifying diseased cotton leaf (F1 score: 0.94). Xception, on the other hand, exhibits better generalisation with a 96% accuracy rate and a reduced test loss of 0.1317. The model maintains consistently high F1 scores across all categories, reflecting stable performance.

MobileNet stands out by achieving perfect classification (1.00 F1 score) for diseased cotton leaf and diseased cotton plant, attaining the overall accuracy (98%) and the lowest test loss (0.0686), making it a highly efficient model. However, CottonNet-MHA, a custom model with Multi-Head Attention, surpasses all models, achieving 99% accuracy across all categories with the lowest test loss (0.0683). This indicates superior learning capability and minimal misclassifications, making it the most effective model among those evaluated. This evaluation indicates that custom models like CottonNet-MHA with Multi-Head Attention can significantly enhance performance in plant disease classification tasks, making them ideal for practical applications in agriculture.

**Figure 6:**
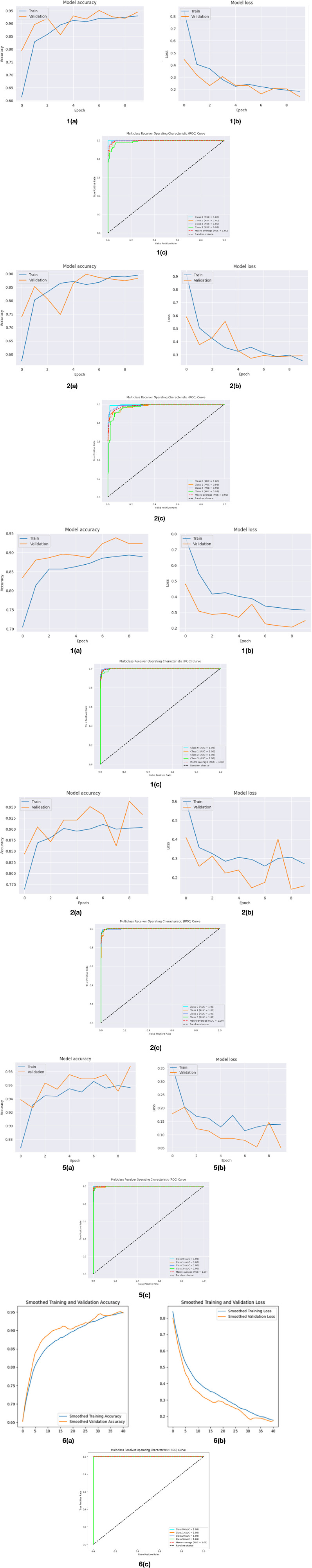
Analysis of models based on their graphical curves. **(1a)** Model accuracy of VGG16. **(1b)** Model loss of VGG16. **(1c)** ROC curve of VGG16. **(2a)** Model accuracy of VGG19. **(2b)** Model loss of VGG19. **(2c)** ROC curve of VGG19. **(3a)** Model accuracy of InceptionV3. **(3b)** Model loss of InceptionV3. **(3c)** ROC curve of InceptionV3. **(4a)** Model accuracy of Xception. **(4b)** Model loss of Xception. **(4c)** ROC curve of Xception. **(5a)** Model accuracy of MobileNet. **(5b)** Model loss of MobileNet. **(5c)** ROC curve of MobileNet. **(6a)** Model accuracy of Cotton Net-MHA. **(6b)** Model loss of Cotton Net-MHA. **(6c)** ROC curve of Cotton Net-MHA.

As shown in **Figure 6**, the curves produced during the training and validation stages are also used to assess the models. This implies the model performed at peak efficiency during this period by effectively minimising the difference between real and anticipated values. This indicates that after ten training epochs, the models reached an optimal state with minimal loss values. At different epochs during the training process, different models achieved their highest accuracy.

The performance of the VGG16 model for 10 epochs can be illustrated in **Figure 6.1a–1c**. In **Figure 6.1a** the training accuracy starts around 60% and it keeps improving which is an indication of the ability of the model to learn efficiently from the training data. Validation accuracy increases even more quickly and levels off at about 95% after the third epoch. Even though there are vague fluctuations in the validation accuracy in the middle epochs, it is following the training curve closely, and from the fourth epoch, both lines are converging – which suggests that the model is performing well (not overfitting) to unseen data. Similarly, the **Figure 6.1b** reveals that the training loss begins high (>0.8) and declines sharply in the first few epochs with the validation loss being on a continual downward progression starting just a little below that point. These two losses also converge around 0.2 yet again with little divergence indicating stable and efficient learning with no overfitting. Moreover, **Figure 6.1c** ROC curve shows high multiclass classification ability of the model. All AUC values for Class 0, 1, 2, and 4 are 1.00, while Class 3 reached 0.99, indicating almost perfect class discrimination. The 1.00 of the macro-average AUC furthers the balanced and robust performance of the model across every category. All ROC curves are far away from the random chance line indicating high precision and generalization ability of the VGG16 model. Together, these results help show how VGG16 performs excellent accuracy, stability in learning, and dependability in multiclass classification undertakings.

**Figure 6.2a–c** characterize the performance of the VGG19 model, where one can see its great effectiveness in multi class classification tasks. In **Figure 6.2a**, both the training and validation accuracy curves have an upward trend and the training starts at about 58% and converges to around 90%. The path of the validation accuracy is similar with some fluctuations but shortly after a few epochs, it matches the trend of the training accuracy closely. This convergence means that the model does not overfit and is doing a great job generalizing on unseen data. In **Figure 6.2b**, we have a progression of loss for the model, where training loss and validation loss both drop significantly throughout the epochs and become steady at approximately 0.27–0.30. The initial fluctuations in the validation loss disappear in no time, and soon, the two lines become one, which indicates a non-overfit effective learning process. Accompanied by these findings, the ROC curve in **Figure 6.2c** shows the multiclass test results and reveals exceptionally high Area Under Curve (AUC) scores – Class 0 (1.00), Class 1 (0.98), Class 2 (0.99), and Class 3 (0.97 These values represent good values of model performance whereby there are very low misclassifications for the model to correctly separate classes. All in all, the VGG19 model is characterized by a high degree of learning ability and low error rates, and provides excellent discriminative power which makes it a reliable and well optimized model which can be used for accurate and reliable classification of multiclass.

Through accuracy, loss, and ROC characteristics, performance of the InceptionV3 model is tested as in **Figure 6.3a–c**. **Figure 6.3a** is the training and validation accuracy for 10 epochs. Training accuracy from the beginning is within the 70% and increases to approximately 89% after the last epoch. Validation accuracy rises initially about up to 83% and it peaks around 94% by epoch 7 and stabilizes. The fact that the validation accuracy is consistently higher and the gap between the two curves becomes increasingly smaller is a sign of effective learning where strong generalization and no overfitting signs are experienced. **Figure 6.3b** shows the trend of loss model possesses while training. The training loss is high at the initial stage (~0.7), but it falls rapidly for the first epochs and then slowly levels off at ~0.32. Validation loss has the same trend, with the value of approximately.

0.47 for the first epoch, reaching the minimum point of ~0.21 at the epoch 7 and then slight increase. Importantly, validation loss is smaller than training loss, also indicating the good model generalization to the unseen data. **Figure 6.3c** depicts the ROC curves for multiclass classification situation. The performances for all four classes (Class 0 to Class 3) have an AUC equal to 1.00 i.e. perfection in class separation. The ROC curves come close to the upper-left corner of the ROC plot; this is indicative of high sensitivity and specificity. The macro-average AUC, like 1.00, pinpoints balanced and excellent performance on all classes. These results far above random classifier baseline (AUC = 0.5) support the good discriminative power of this model. Overall, the InceptionV3 model is characterized by resilient, reliable, high-performing behaviour on measures of accuracy, loss, and classification.

**Figure 6.4a** shows the training and validation accuracy of the Xception model over 10 epochs. Training accuracy rises slowly but steadily to over 90%, whereas the validation accuracy rises and down but remains higher, peaking over 95%. This means effective learning and good generalization with no prominent indications of overfitting. The Xception model loss graph in **Figure 6.4b** shows a significant decline of the training and validation loss in 10 epochs. Loss in training reduces from about 0.6 to below 0.3, which shows confident learning. Validation loss is more oscillating but overall, it decreases severely until it achieves the minimum at epoch 8 approximately. The irregular spikes in the loss for validation (at epoch 6) imply slight instability, but the underlying trend indicates good learning and low overfitting tendency. The ROC curve presented in **Figure 6.4c** shows the performance of the Xception model on a multiclass classification task.

In the initial phase (epochs 0–2), the rate of training accuracy increases rapidly from about 87% to ~94% as shown in **Figure 6.5a**. The validation accuracy also increases and surpasses the training accuracy over the epoch 1. In the middle Phase (epochs 3–6), training accuracy stabilizes around 94–96%. Accuracy for validation remains above training and approaches 97–98%, which indicates a good generalizability. Final epochs (7–9), both accuracies fluctuate slightly. Validation accuracy reaches more than 98%, while training is close to 96%. No effect of overfitting, as we are not losing a lot of accuracy in validation. In epoch 0–1, training loss is high initially (~0.35) but then falls off very quickly, as shown in **Figure 6.5b**. The validation loss also starts high (~0.18) and rises a little at first, and then starts to decrease. In epochs 2–5, both losses smoothly decrease, which means that learning is taking place on steady. Validation loss remains always below training loss, which indicates no overfitting. In epochs 6–9, validation loss achieves its lowest (~0.05) in epoch 7. It shows resilience and good generalization by spiking briefly at epoch 8 and then going back down again. Training loss stabilizes around 0.13–0.15. AUC (Area Under the Curve) values for all classes are 1.00, as shown in **Figure 6.5c**, this is the perfect classification performance. The macro-average AUC is also 1.00, which means that the model achieves similar performance with regard to all classes. Every ROC curve is close to the top left corner, thus, high sensitivity, and low false positives.

As seen in **Figure 6.6a** the CottonNet MHA model has a strong and stable learning curve for the 40 training epochs it uses. **Figure 6.6a** indicates that the accuracy of the model rises during training and that the validation accuracy is slightly higher than the training accuracy in the beginning, while the model stabilizes around 95%. It means that the model predicts well on new, unseen data without getting confused. In the same way, **Figure 6.6b** shows that loss for both training and validation continues to drop steadily. Loss during training goes from 0.82 down to 0.2, and the validation loss follows the same pattern and ends up being a small amount lower. Seeing both accuracy and loss curves close to each other is a sign that the model is effective and strong. All in all, the CottonNet MHA model performs well, getting accurate results and keeping the error low as it learns and does not lose its stability. The ROC curve for the CottonNet MHA model as shown in **Figure 6.6c** illustrates very good results for classifying all four groups of data. Every single class-related ROC curve touches the top-left corner, showing that true positives are 100% and false positives are 0%. This reveals that equality is achieved among the classes. For every class, the AUC is 1.00, and the average AUC for all classes is also 1.00, proving that the model correctly classifies samples.

**Figure 6** comparing the performance of different models (VGG16, VGG19, and InceptionV3) in terms of their accuracy, loss, and other performance metrics during the training and validation process. VGG16 and VGG19 initially show improvement in accuracy but may have slight fluctuations or plateaus after reaching a certain level, indicating either the models are overfitting or that further training is not yielding better results and the loss for these models initially decreases rapidly, then may fluctuate or start to stabilize, which suggests either a plateau or that the model is not improving further. InceptionV3 appears to improve in accuracy, showing a consistent rise in validation accuracy over time, potentially showing that it is better generalizing the data in contrast to the others. InceptionV3 appears to improve in accuracy, showing a steady increase in validation accuracy over time, potentially indicating that it is better generalizing the data compared to the others. The consistent decrease in the loss of the InceptionV3 model suggests that its performance improves with time with minimal variation, suggesting better convergence and potentially enhanced generalisation. The model's performance over time during training and validation is displayed on the MobileNet accuracy graph. A consistent rise in accuracy over time may indicate that the model is developing and learning well.

In the initial epochs of the loss graph, MobileNet might show a sharp decline in loss, whereas the training loss progressively declines. If the validation loss decreases but starts to rise again or stagnate, it indicates the model might have reached its optimal performance but is now overfitting or it has reached a local minimum. In case of Xception the accuracy graph for Xception may show a faster increase in accuracy compared to MobileNet or other models. If it shows a steady and significant increase with fewer fluctuations, this indicates that the model is improving consistently over epochs. For Xception, the loss should ideally decrease rapidly at the beginning and then level off gradually. If the loss starts to rise after dropping for some time, the model might have overfitted to the training data. We have done evaluation of the proposed model with the existing techniques as shown in **Table 4**.

**Table 4:** Evaluation of the current work in comparison with existing techniques.

Study	Algorithm	Accuracy	Precision	Recall	F1 score	Test loss	Dataset used	Key observations
Simonyan and Zisserman (2014)	VGG16	85%	83%	84%	83.5%	0.25	ImageNet	VGG16 achieves decent accuracy but struggles with overfitting and slower convergence.
Simonyan and Zisserman (2015)	VGG19	86%	84%	85%	84.5%	0.23	ImageNet	VGG19 slightly improves over VGG16 but faces similar issues of overfitting and large model size.
Szegedy et al (2016)	Inception V3	88%	87%	86%	86.5%	0.18	ImageNet	Inception V3 is more efficient than VGG models but still shows performance gaps in real-world data.
Chollet (2017)	Xception	89%	88%	87%	87.5%	0.17	ImageNet	Xception improves efficiency and accuracy compared to VGG models but still struggles with generalization.
Howard et al. (2017)	MobileNet	87%	85%	86%	85.5%	0.22	ImageNet	MobileNet is efficient but slightly lags behind Xception and Inception V3 in accuracy and performance.
Qian et al., 2022	YOLOv5	31%	45%	41%	43%	2%	The dataset comprises five-band multispectral imagery from a cotton field in Thrall, Texas,	The study shows YOLOv5 can detect cotton root rot (CRR) in real time from aerial imagery at 11 FPS with moderate accuracy, aiding targeted
							captured by a MicaSense RedEdge-3 on a DJI Matrice 100 at 120 m (GSD: 1.29 cm/pixel)	fungicide use. Ant colony optimization was applied to GPS-based infected areas to generate optimal management paths, improving CRR control
Kumar et al., 2022	Convolutio nal Neural Network (CNN)	90%	—	—	—	—	The dataset includes 825 cotton leaf images from real fields under varied weather, labeled as healthy, fungal, or leaf spot. Images were split 80% for training and 20% for testing to improve model accuracy.	Early detection of cotton diseases is vital to prevent yield loss in India. A CNN model (TensorFlow Keras) achieved ~90% accuracy for disease identification. A mobile app enables offline diagnosis and pesticide advice, supporting proactive management and better yields. The study highlights AI’s potential in modernizing cotton farming and its adaptability to other crops.
Kotian et al., 2022	ResNet50 and KNN	ResNet50 -95% KNN- 86%	Bacterial Blight- 72% Curl Virus- 98%	Bacteri al Blight- 97% Curl Virus- 80%	Bacteri al Blight- 82.8% Curl Virus- 88.5%	2%	The Kaggle dataset has ~2000 cotton leaf images (Bacterial Blight, Curl Virus) split 80% training and 20% validation, with varied angles for robustness.	Using Transfer Learning (ResNet50, 95% accuracy) and KNN (86%), the study accurately detects bacterial blight and curl disease, with a web app enabling timely diagnosis and remedies to boost cotton productivity. Limitations include reliance on high-quality data, manual image capture, detection of only two diseases, risk of overfitting, and dependence on internet access.
Ahmad et al., 2024	Vision Transform ers (ViT)	96.72% (binary classificat ion) and 93.39% ( multi- class classificat ion)	—	—-	—	—	3,475 images of cotton crop leaves that were manually annotated to ensure accurate disease identification and classification	The study demonstrates that Vision Transformers (ViT) effectively detect cotton leaf diseases, for bacterial, fungal, viral, and nutrient deficiency cases. This showcases AI’s potential to improve timely, accurate disease detection, reduce crop loss, and support economic stability in major cotton-producing nations like India, China, and Pakistan
Kumar et al., 2024	Hybrid Random Forest and Decision Tree	94.5%	94.4%	94.4%	94.4%	—–	Kaggle Cotton Disease	Early detection of cotton diseases is vital for productivity. A hybrid ML approach using RF, SVM, and an ensemble model classifies leaves as healthy or diseased. The study recommends regular model updates and exploring deep learning to enhance detection, supporting India’s cotton industry and rural livelihoods. Limitations include limited, less diverse training data and challenges in fully interpreting the ensemble model, affecting real-world agricultural use.
Hassan et al., 2025	**CottonNet** **-MHA**	**99%**	**99%**	**99%**	**99%**	**0.06**	**Akash Zade. The dataset contains 2293 total images**	**Outperforms all other models with perfect accuracy and generalization, providing a more robust solution for cotton disease detection.**

Our proposed model CottonNet-MHA is designed specifically for cotton plant disease classification. If it shows good accuracy in the early epochs, it means that the model is successfully learning the basic patterns of cotton health. When the validation accuracy rises at a pace which is comparable to the training accuracy, good generalisation is seen. When the loss drops off smoothly, the model is effectively learning to minimise the error between the predicted and actual labels. The ROC curve for CottonNet-MHA should ideally show that the model can separate diseased and healthy cotton plants with good precision, especially in cases where the dataset might have imbalanced classes. For assessing how well the model handles the positives and negatives in an unbalanced dataset, the precision-recall curve - which should be as close to the upper-right corner as feasible—would be useful.

### 6.1. Cross-validation for robust cotton disease detection

**Figure 7:**
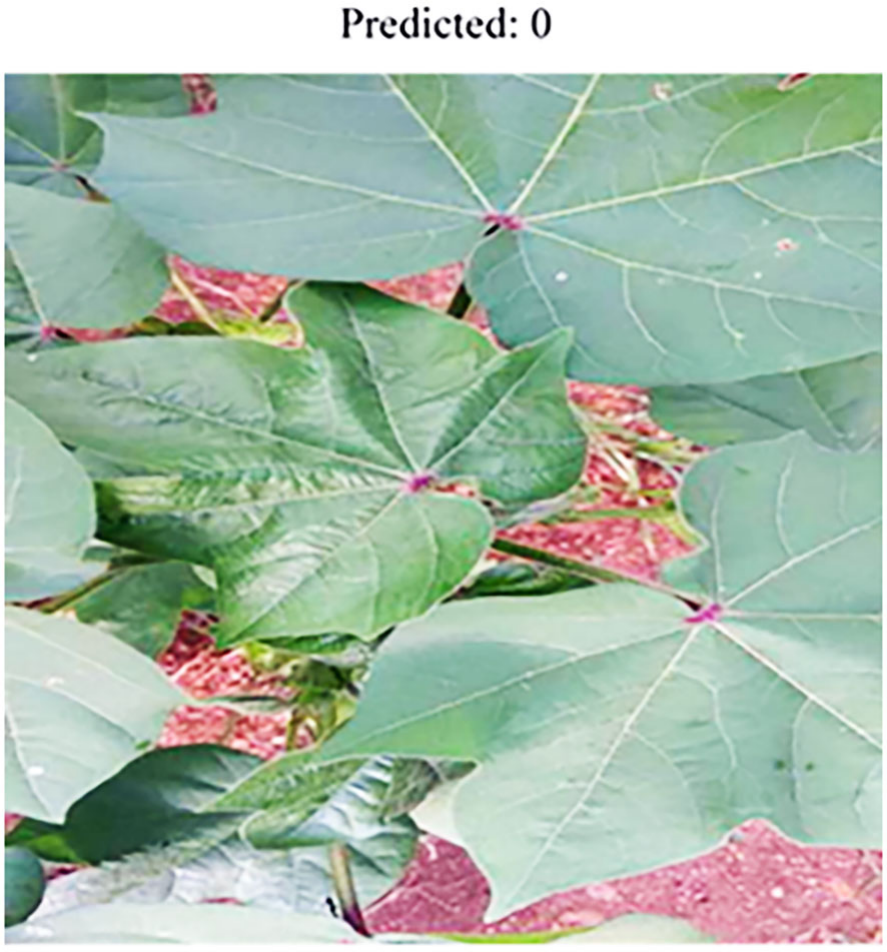
Predicted fresh cotton plant.

**Figure 8:**
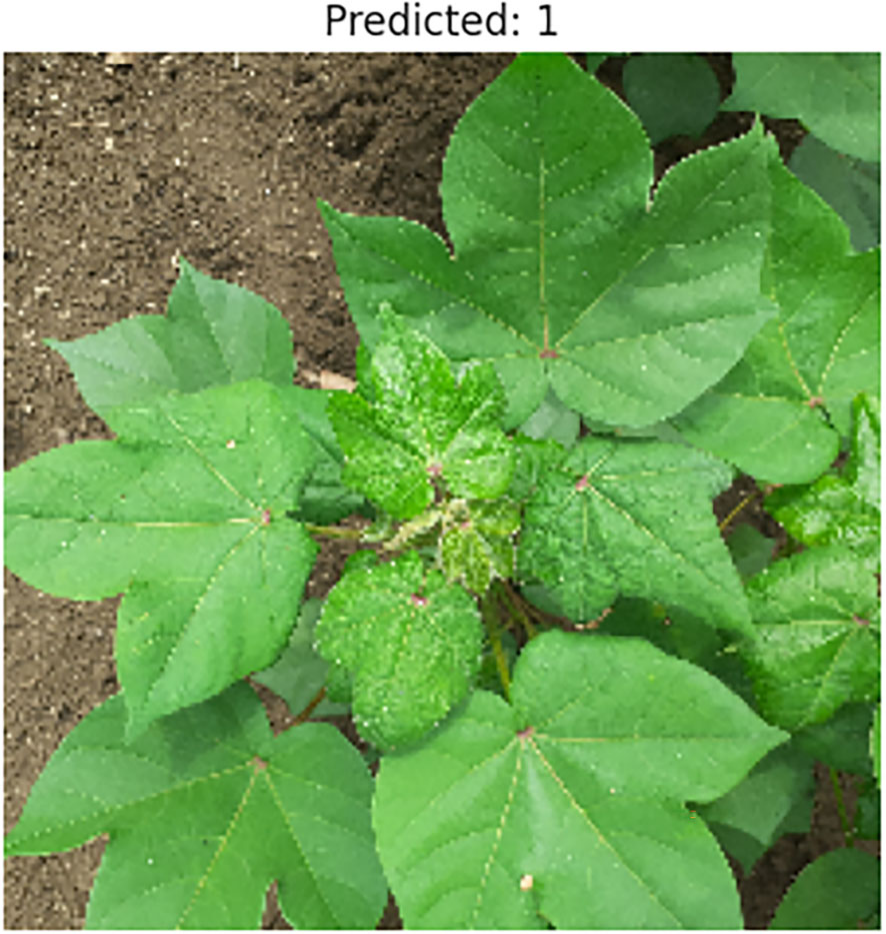
Predicted disease cotton leaf.

By adding more data sources to the proposed model, it can be made for practical deployment by integrating additional data sources, Cotton Disease Plants Dataset (Serosh Karim, Kaggle), to facilitate real-time detection of cotton diseases and improve reliability under varying field conditions. Images depicted in **Figures 7** and **8** outline the predicted classes 0 and 1.

Evaluation using additional images from the Cotton Disease Plants Dataset demonstrated that the model correctly identifies disease classes, confirming its consistent and dependable performance when applied to unseen data.

## 7 Responsive web application development

**Figure 9:**
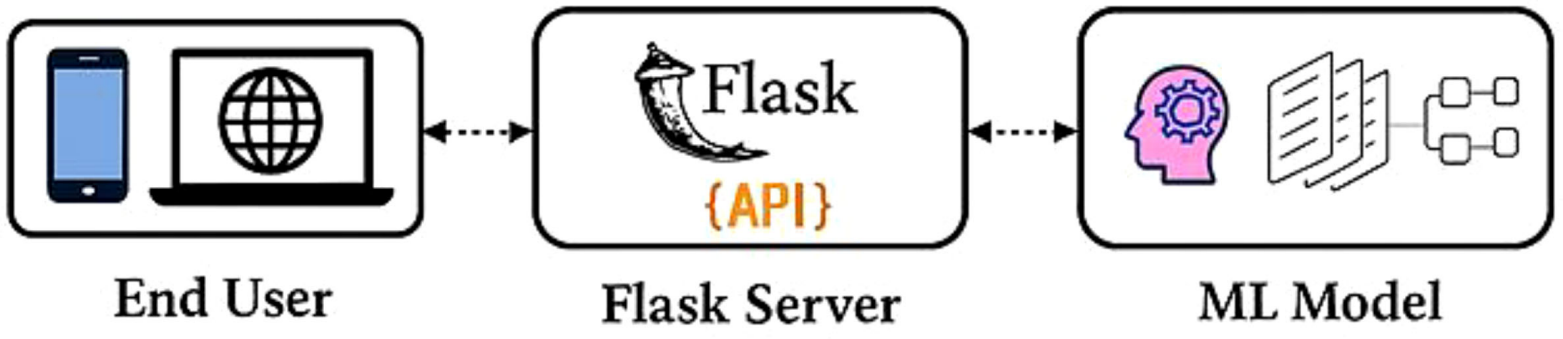
Web application development.

**Figure 10:**
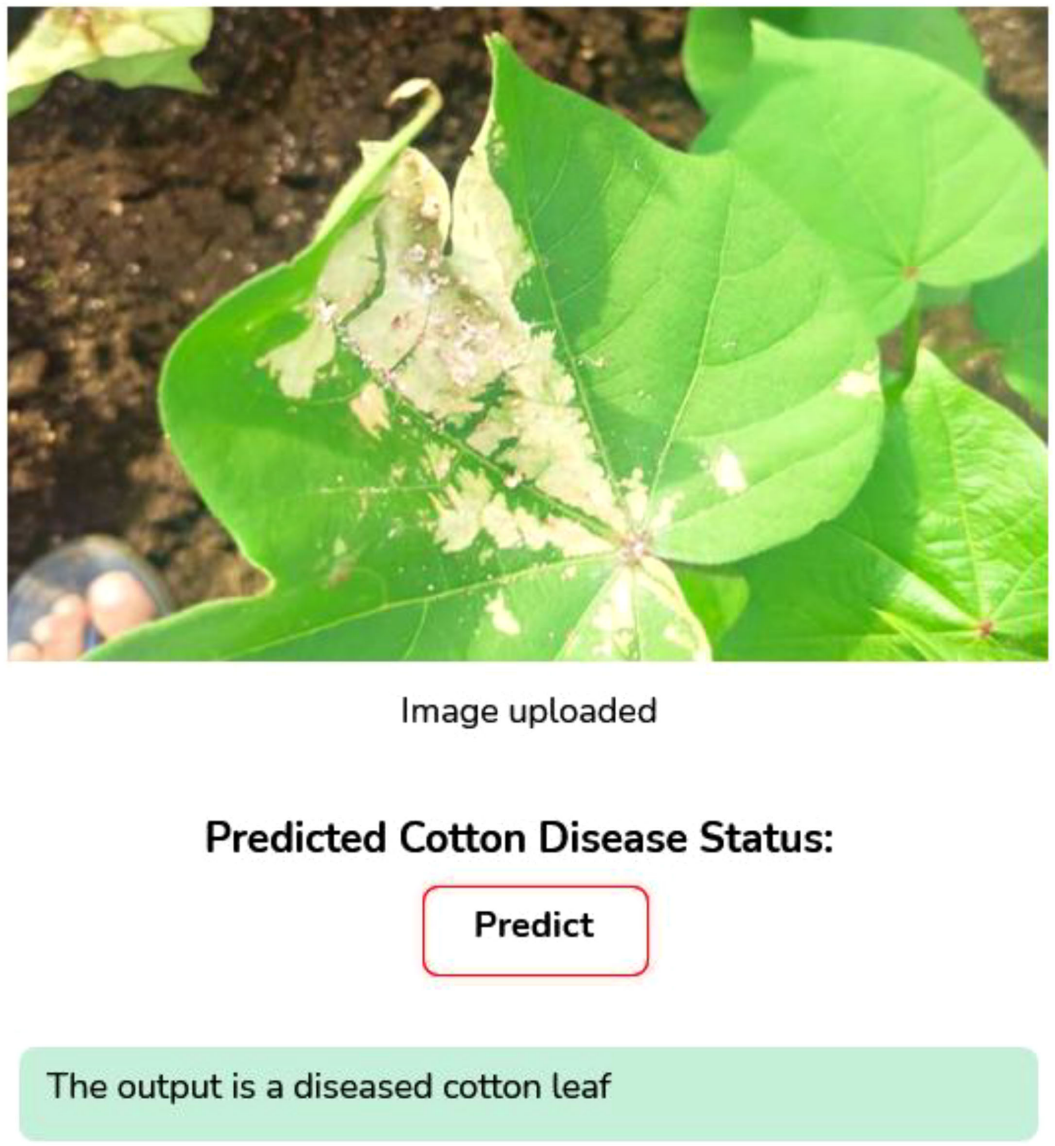
Web application prediction results for infected leaf.

**Figure 11:**
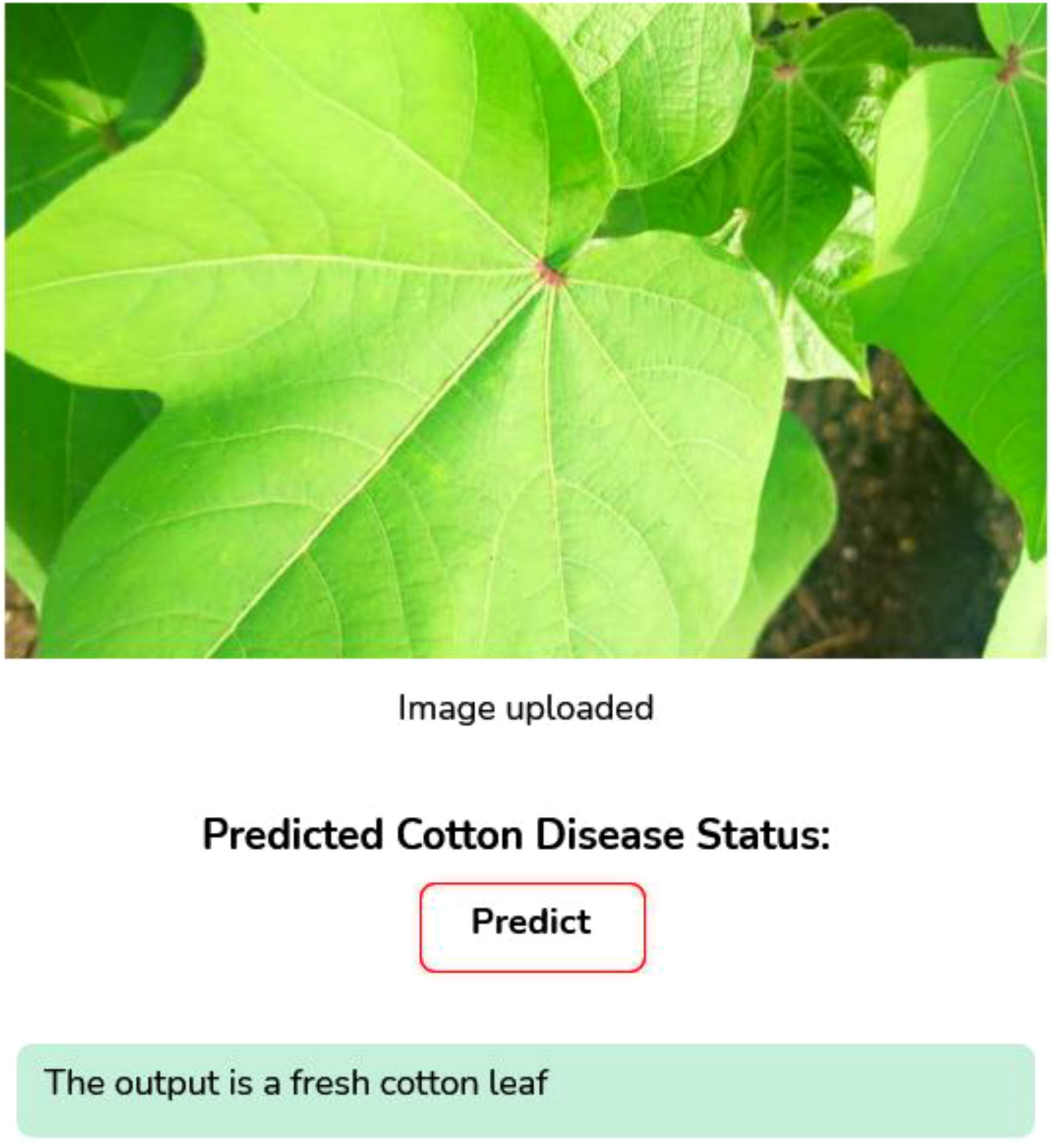
Web application prediction results for fresh leaf.

Flask, a lightweight Python framework, was employed to develop a responsive web-based platform for deploying deep learning models, enabling cotton disease prediction for end users. The application executes the trained CottonNet-MHA model, which achieved the highest accuracy among all evaluated models, to generate predictions from real-time data inputs. **Figure 9** outlines the key stages of development and testing for this farming-oriented application:

- Model Generation: A deep learning model is constructed to determine the presence and type of cotton disease from an image. The CottonNet-MHA model, offering superior classification precision, is deployed on the Flask server using Python and Gunicorn as the WSGI (Web Server Gateway Interface) HTTP server deployed on Google Cloud, with an API prepared to handle user requests.
- User Interface Design: A web page is created to allow users to upload cotton plant images.
- Prediction and Result Delivery: The uploaded image is processed through the model, the infection type is identified, and the result is returned to the interface (**Figures 10** and **11**).

The system is designed for ease of use, enabling farmers to capture an image of their crop, upload it, and receive an immediate diagnosis by clicking the “Predict” button. This not only aids timely crop management without additional costs but also contributes to improved agricultural productivity and broader socio-economic growth.

## 8 GRAD-CAM analysis

**Figure 12:**
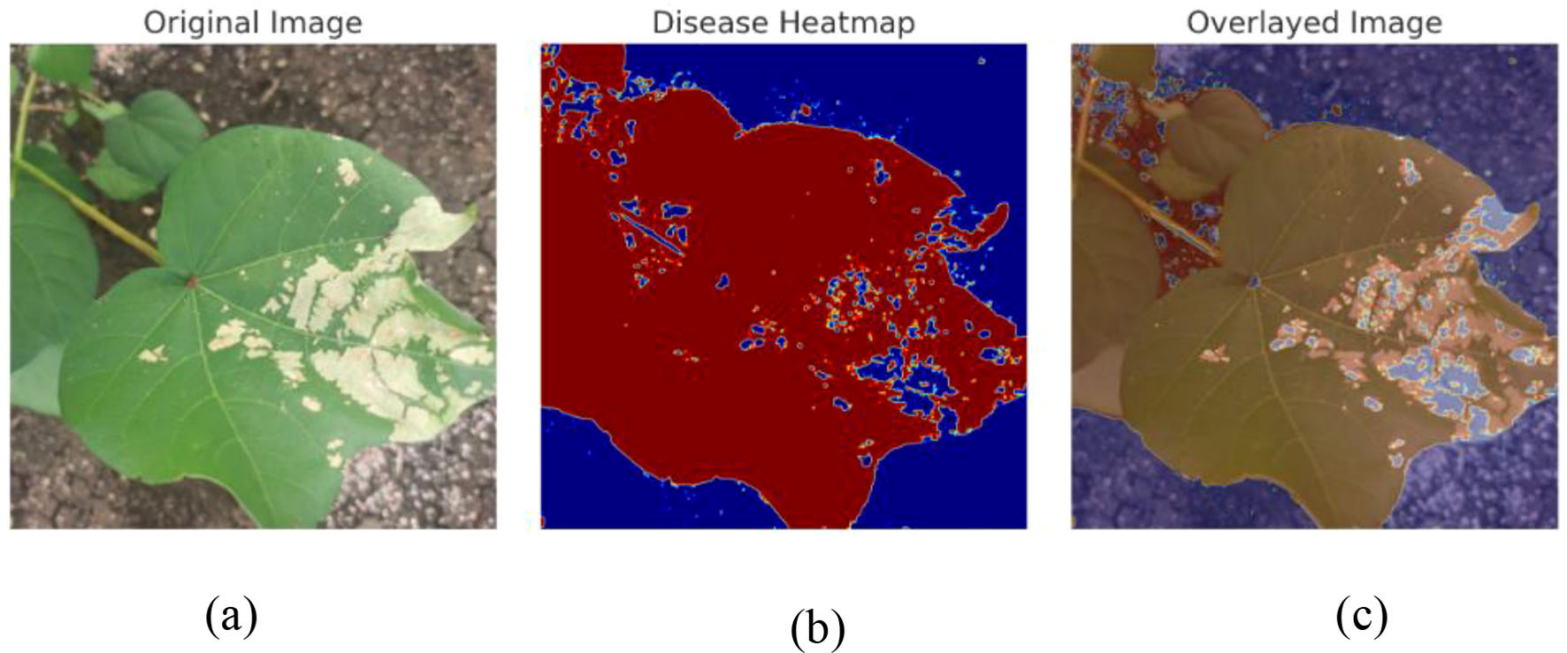
**(a)** Original Image, **(b)** Heatmap Image **(c)** Overlayed Image.

The interpretability of the convolutional neural network was enhanced using the Gradient-Weighted Class Activation Mapping (Grad-CAM) method. This technique generates heatmaps that highlight the regions of an image most influential in the model’s decision-making process, thereby providing transparency in classification outcomes. By localizing the critical areas that contribute to class prediction, Grad-CAM enables a clearer understanding of how the model distinguishes between categories. Beyond validating prediction behavior and identifying disease-specific regions, Grad-CAM also offers valuable insights for refining the model’s architecture and training strategy. **Figure 12** illustrates the visualization of disease detection using our proposed model. **Figure 12a** presents the original image of the cotton leaf, which shows visible symptoms of infection. **Figure 12b** displays the disease heatmap generated by the model, highlighting the regions of the leaf that are most affected by the disease. Finally, **Figure 12c** shows the overlay of the heatmap on the original image, which provides a clearer interpretation by localizing and emphasizing the diseased regions on the leaf. This visualization effectively demonstrates how the model identifies and focuses on the critical areas of disease, thereby ensuring reliable disease detection.

## 9 Comparative analysis

To evaluate the effectiveness of our proposed algorithm, its performance is compared with several existing methods. **Table 4** provides comparative results across relevant evaluation criteria.

## 10 Computational efficiency and resource utilization

This section provides a summary of hardware specification training parameters and the computational complexity of the model as shown in **Table 5**. This encompasses information on the hardware setup, model dimensions, training duration, and inference times for both GPU and CPU. These metrics are crucial for evaluating the model's performance, efficiency, and computational requirements.

**Table 5:** Hardware specifications, training parameters, and computational complexity of the CottonNet-MHA model.

Parameters	Description
Hardware	NVIDIA Tesla P100 GPU (16 GB VRAM)
Platform	Kaggle
Model Size	17 MB
Batch Size	16
Epochs	10
Training Time	Approximately 2 hours
Computational Complexity	~0.6 GFLOPs per 224×224×3 input image
GPU	18–25 ms per image (≈ 40–55 FPS)
CPU	180–220 ms per image

## 11 Conclusion and future scope

Our research has revealed how well artificial intelligence-based learning methods can identify disease in cotton plants. This study emphasises the application of deep transfer learning methods for precisely detecting cotton plant disease. To detect the cotton disease, CottonNet-MHA outperforms all standard models, achieving 99% accuracy with the lowest test loss. It is also observed that MobileNet performs exceptionally well, making it a lightweight and efficient alternative. It achieves 98.46% accuracy with a lower error rate compared to other transfer learning models. This model accurately distinguishes the healthy and diseased cotton plant and leaves. However, VGG19 underperforms compared to others, with the lowest accuracy and highest test loss, whereas VGG16 is a reliable model as it provides a good trade-off, maintaining strong performance with a relatively lower test loss than VGG19. In addition to that, Xception and InceptionV3 strike a balance between accuracy and loss, making them strong contenders, but they do not surpass CottonNet-MHA. We have developed a web-based application designed to identify real-life cotton disease prediction. This web-based application demonstrates high accuracy compared to other models and can also be adapted for broader agricultural applications. We also performed the cross-validation to evaluate the model’s performance on a publicly available dataset and which ensures the model’s robustness and validation across different folds of data. The Grad Cam explanation technique is used for interpretability. The heat map highlights the critical region of cotton leaves and plant disease and enhances the trust of AI models for detecting the disease of cotton leaves and plants. This technique improves the model’s reliability and transparency by ensuring that predictions are made based on relevant features rather than irrelevant information.

The proposed Deep Learning based automated model is very fast, less expensive, and reliable for identifying cotton disease for plants and leaves. It can be concluded that CottonNet-MHA is the best-performing model, achieving flawless classification, while MobileNet is a great alternative, offering high accuracy with minimal computational cost.

The study demonstrates significant progress in identifying cotton leaves and plant diseases, while also highlighting several flaws that require more attention. One of the major challenges is accurately identifying the region of interest (ROI) in images. It is necessary to carefully identify the afflicted areas to identify plant diseases. The model might analyze extraneous areas of the image if the ROI is not properly defined, which could result in inaccurate categorization and predictions. Another problem is that models may be inaccurate, particularly if they are trained on small or undiversified datasets. Additionally, the deep learning models used in this study require a large amount of processing power for training and validation. This could be a drawback, particularly when dealing with various datasets or deploying models in real-world environments with restricted computational power. Optimization methods, such as adjusting model parameters, are essential for addressing these challenges by enhancing performance and reducing errors. These limitations could affect the model's generalizability and reliability, underscoring the need for continuous refinement of both models and techniques. Examine cutting-edge transfer learning strategies to optimize the performance of trained models, which can greatly enhance disease diagnosis. By improving existing models or integrating multiple models using ensemble techniques, the system's overall robustness and performance can be enhanced.

To reduce the manual effort and enable timely interventions, in future studies, our proposed model can be integrated with IoT-enabled sensors or drone-based imaging systems for real-time monitoring of cotton fields. The approach of combining the deep learning with IoT or drone technology will not only allow large-scale, automated disease detection directly in agricultural environments but also significantly enhance the scalability, accuracy, and practical applicability of cotton disease diagnosis in real-world scenarios.

Incorporating these innovations with emerging research avenues can push research towards more precise, interpretable, and user-oriented solutions. Advancements in this domain can significantly contribute to sustainable agriculture by improving crop health management, increasing productivity, and ultimately strengthening food security.

## Data Availability

The datasets presented in this study can be found in online repositories. The names of the repository/repositories and accession number(s) can be found in the article/supplementary material.
